# Microplastic and lead shift microbiomes enriching viral auxiliary metabolic genes for potential polylactic acid degradation

**DOI:** 10.1038/s42003-026-10162-7

**Published:** 2026-05-07

**Authors:** Xieluyao Wei, Kinza Bashir, Xianrui Tian, Akasha Farooq, Expedito Olimi, Tomislav Cernava, Lingzi Zhang, Xiumei Yu, Qiang Chen, Petri Penttinen, Yunfu Gu

**Affiliations:** 1 College of Resources, Sichuan Agricultural University, Chengdu, China; 2 School of Biological Sciences, Faculty of Environmental and Life Sciences, University of Southampton, Southampton, UK; 3 Institute of Environmental Biotechnology, Graz University of Technology, Graz, Austria

**Keywords:** Virology, Metagenomics

## Abstract

Biodegradable microplastics and heavy metals increasingly co-occur in soils through plastic mulching, organic amendments, and legacy metal contamination. Yet, their combined effects on soil–plant–microbiota interactions remain unclear, particularly for the virus. Here we evaluated the impacts of bio-MPs, polylactic acid (PLA), lead (Pb), and their combination on buckwheat and rhizosphere bacterial-viral communities. Co-contamination reduced soil pH and nutrient availability, increased Pb accumulation in plant tissues and suppressed buckwheat growth. Metagenomic analyses revealed that both bacterial and viral communities were altered under Pb-containing treatments. Bacterial genes associated with carbon and phosphorus metabolism were suppressed, while viral auxiliary metabolic genes (AMGs) related to carbon utilization were enriched, especially carbohydrate esterases that hydrolyze PLA ester bonds. A putative AMG-associated carbohydrate esterase gene (P9222_28545) was identified and the esterase activity confirmed via heterologous expression in *E. coli*. These findings highlight a potential role of viruses in mediating microplastic degradation in soils.

## Abstract (web summary)

PLA–Pb co-contamination disrupts soil–plant-microbiome interactions and suppresses plant growth, while enriching viral AMGs, including carbohydrate esterases, that may facilitate bioplastic degradation and reshape rhizosphere microbial functions.

## Introduction

Biodegradable plastics are increasingly developed to address the growing concern over plastic pollution, with polylactic acid (PLA) being the most widely produced biodegradable plastic globally^1^. As PLA degrades, it can fragment into microplastics (MPs, particles <5 mm), which tend to accumulate in terrestrial soil^2^. Despite being classified as biodegradable, PLA degrades extremely slowly under natural environmental conditions, and thus PLA-MPs pollution can be an emerging threat. After entering soil, MPs can act as passive samplers and vectors for heavy metals, as their negatively charged surfaces and diverse functional groups promote the adsorption of metal ions onto MP surfaces^3,4^. Growing evidence suggests that the interaction between MPs and heavy metals can reduced microbial richness, diversity, and metabolic functions^5,6^. Although previous research has explored bacterial community responses to such co-contaminations^4,7^, less is known about how soil viral communities respond to these complex environmental stresses.

Viruses are the most abundant and diverse biological entities on Earth and have been detected in nearly all explored ecosystems^8^. Through lytic and temperate lifestyles, viruses influence microbial population dynamics, nutrient turnover, and energy metabolism^9–11^. Beyond direct lysis, viruses affect host metabolism via auxiliary metabolic genes (AMGs), which can enhance bacterial survival and ecological functioning^12^. In soil ecosystems, viral abundance can reach up to 10⁹ particles per gram^13^, yet their ecological roles, especially under anthropogenic stresses, remain underexplored. Recent studies suggest that viruses modulate microbial adaptation by regulating host densities, transferring genes, and encoding stress-responsive AMGs^14,15^. In contaminated environments, metal(loid) stress can alter viral diversity, shifts in lifestyle strategies, and enrichment of stress-related AMGs^16,17^. Given that viral AMGs can extend host metabolic capacities, viruses may potentially encode enzymes capable of facilitating PLA depolymerization and contributing to PLA degradation. However, how viral AMGs functionally respond to co-contamination with MPs and heavy metals remains poorly understood. In particular, it remains unknown whether viruses encode AMGs associated with the degradation of biodegradable MPs (bio-MPs) such as PLA. Given their distinct lifestyles, bacteria and viruses may exhibit divergent functional responses to environmental stress; bacteria being more directly inhibited, while viruses may expand their functional repertoire via AMG-mediated strategies^12,18^.

Soil lead (Pb) pollution is widespread in China, with national-scale assessments reporting a weighted mean concentration of 35.9 ± 0.21 mg kg⁻¹ in surface soils over the past three decades and more severe contamination in southern regions^19^. In parallel, MPs contamination in croplands has increased markedly, with average concentrations rising from ~100 items kg⁻¹ soil in 1980 to over 2000 items kg⁻¹ soil by 2018, and higher levels generally observed in southern China. With the growing adoption of biodegradable plastic films, biodegradable polymers such as PLA are expected to become an increasingly relevant component of soil MPs pools in the future^20^. Buckwheat (*Fagopyrum tataricum* L.), an important economic and food crop widely cultivated in southern China, its well documented to tolerate accumulate of heavy metals and has therefore been widely studied as a model crop under metal stress; however, its response to MPs exposure remains largely unexplored^21^. Our previous study showed that PLA-MPs, especially when combined with Pb, induced stronger toxicity to buckwheat and more severe reductions in rhizosphere bacterial diversity than nondegradable MPs (non-MPs) such as polyethylene (PE) MPs^6^.

In this study, we investigate the effects of PLA and Pb, individually and in combination, on soil microbial communities in the buckwheat rhizosphere using metagenomic and viromic approaches. We further assess whether virus-associated AMGs with potential PLA degrading activity can be identified and functionally validated. Here we show that PLA and Pb co-contamination alters soil physicochemical properties, suppresses buckwheat growth, reshapes rhizosphere bacterial and viral communities at both taxonomic and functional levels. Bacterial nutrient-cycling functions decrease, whereas viral functional potential increases, including enrichment of AMGs linked to potential PLA degradation, supported by experimental validation of a virus-associated carboxylesterase (CarE) gene.

## Results

### PLA and Pb alter soil nutrients and impair buckwheat growth

After 60 days of incubation, both Pb-containing treatments (Pb and Pb+PLA) exerted significant effects on multiple soil parameters and buckwheat properties, whereas PLA alone showed more selective impacts. Soil pH was lower in the Pb-containing treatments than in the other treatments (*p* < 0.05) (Supplementary Table 1). In comparison to control (CK), soil alkaline hydrolysable nitrogen (AN) content was higher in the Pb+PLA treatment, while the soil organic carbon (SOC) content was lower in the PLA treatment (*p* < 0.05). The available lead (APb) content was higher in both Pb and Pb+PLA treatments compared with the control and was slightly higher in the Pb+PLA than in the Pb treatment (*p* < 0.05). Compared CK, the soil alkaline phosphatase (ALP) activity was higher in PLA and lower in Pb-containing treatments (*p* < 0.05). No significant differences were observed in total nitrogen (TN), available potassium (AK), available phosphate (AP) or acid phosphatase (ACP) among treatments (*p* > 0.05). Two-way ANOVA revealed an interaction effect between PLA and Pb on the ALP activity and SOC content (*p* < 0.05) (Supplementary Table 2).

Buckwheat growth was significantly inhibited under Pb-containing treatments. Compared to CK, shoot length, stem diameter, and both fresh and dry biomass were reduced in the Pb and Pb+PLA treatments (*p* < 0.05) (Supplementary Table 3). The Pb concentration was higher in the Pb+PLA than in the Pb treatment (*p* < 0.05). Malondialdehyde (MDA) content was lower in the Pb and Pb+PLA treatments compared with CK and PLA. Peroxidase (POD) activity was higher in the Pb+PLA than in the other treatments (*p* < 0.05). The superoxide dismutase (SOD) activity or chlorophyll content showed no significant differences among treatments (*p* > 0.05). Two-way ANOVA revealed an interaction effect between PLA and Pb on the buckwheat biomass and POD activity (*p* < 0.05) (Supplementary Table 2).

### PLA and Pb induce shifts in rhizosphere bacterial community

**Fig. 1:**
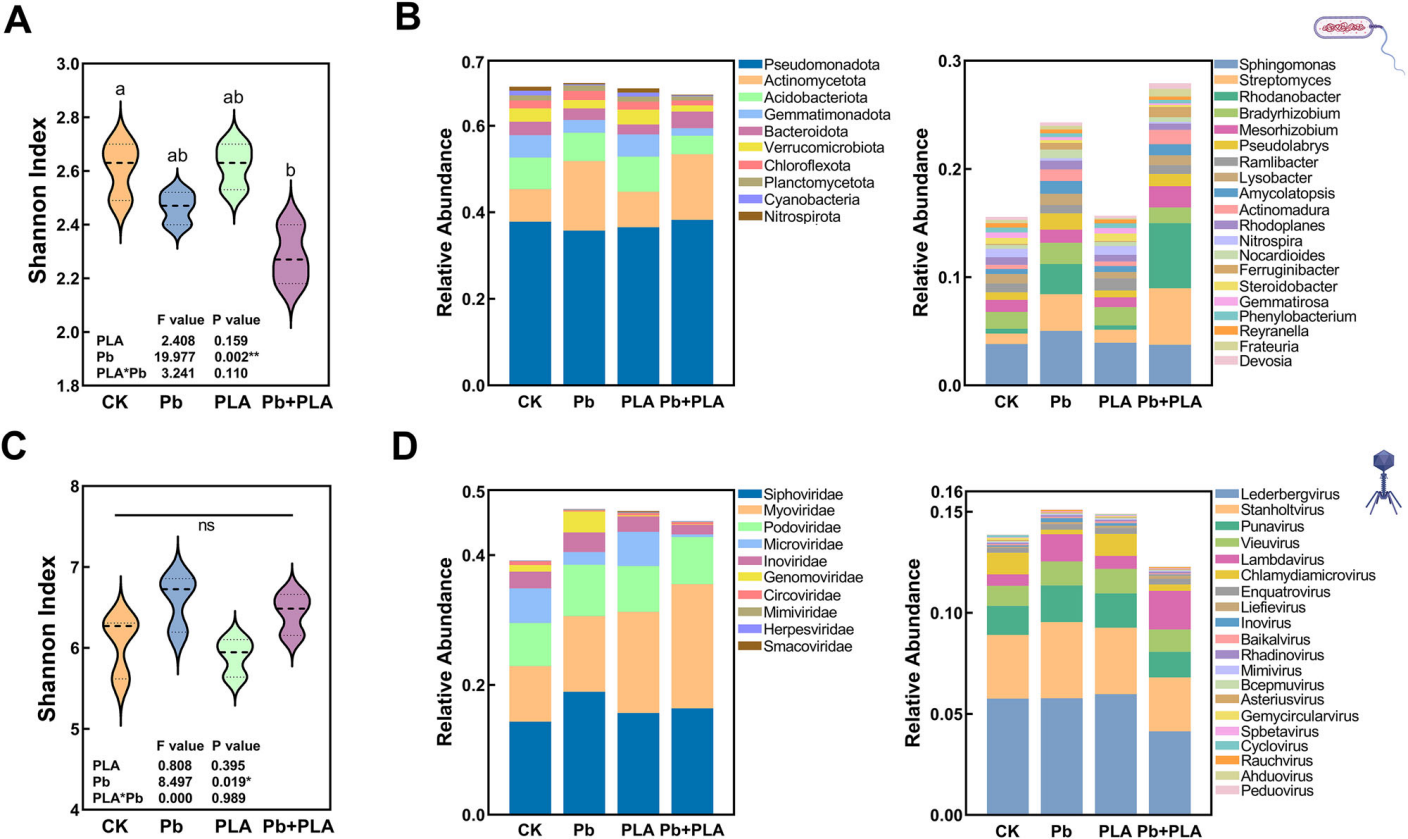
Bacterial and viral communities in buckwheat rhizosphere soil. **A** α-diversity (Shannon index) of bacterial communities, the violin plots show the distribution of the data, with dashed lines indicating the median and quartiles. **B** Relative abundances of the ten most abundant bacterial phyla and the twenty most abundant genera. **C** α-diversity of viral communities. **D** Relative abundances of the ten most abundant viral families and the twenty most abundant genera. CK, control with no PLA-MPs and Pb; PLA, polylactic acid microplastics treatment with 2 g PLA-MPs kg^−1^ soil; Pb, Pb treatment with 2 g Pb kg^−1^ soil; Pb+PLA, Combined Pb and PLA-MPs treatment with 2 g Pb and 2 g PLA-MPs kg^−1^ soil. *n* = 3 biologically independent samples.

Pb-containing treatments were associated with pronounced shifts in bacterial community composition. Metagenomic analysis yielded from 360250 to 494705 contigs per sample (Supplementary Data 2). The Shannon diversity was lower in Pb+PLA compared to the control (*p* < 0.05) (Fig. 1A). At the phylum level, all soil samples were dominated by Pseudomonadota (relative abundance ranging from 35.77 to 38.24%), followed by Actinomycetota (7.50 to 16.08%), Acidobacteriota (4.28 to 8.09%), and Gemmatimonadota (1.74–5.16%) (Fig. 1B). On the genus level, *Sphingomonas*, *Streptomyces*, *Rhodanobacter*, *Bradyrhizobium*, and *Mesorhizobium* were predominant (Fig. 1B).

In comparison to the control, the relative abundances of *Streptomyces* were higher and those of *Nitrospira*, *Ramlibacter*, and *Steroidobacter* were lower in the Pb-containing treatments (*p* < 0.05) (Supplementary Fig. S1). The relative abundances of *Rhodanobacter* and *Ferruginibacter* were higher in the Pb+PLA treatment than in the control (*p* < 0.05). The bacterial community composition differed across all treatments (Adonis *R*² = 0.851, *p* = 0.002) (Supplementary Fig. 2A; Supplementary Table 4), yet the pairwise differences between treatments were not statistically significant. Differences in community composition were associated with differences in APb (adj. *R*² = 13.7%, *p* = 0.001) and AK (adj. *R*² = 17.1%, *p* = 0.047) (Supplementary Fig. 2B; Supplementary Table 5). Fitting all soil physicochemical variables using envfit suggested that pH and APb, AK, TN, and AN content were correlated with community ordination (*p* < 0.05) (Supplementary Table 5).

### PLA and Pb reshape rhizosphere viral community

The assembly of viral reads resulted in approximately 11730 to 38363 virus contigs per sample (Supplementary Table 6). Of these, a total of 7788 contigs were identified as viral sequences using CheckV^22^ and Virsorter2^23^. In contrast to the bacterial community, the viral diversity was similar across all treatments (*p* > 0.05) (Fig. 1C). Among the annotated viruses, viral families such as Siphoviridae, Myoviridae, and Podoviridae accounted for over 75% of the total relative abundances, followed by Microviridae, Inoviridae, Genomoviridae, Circoviridae, Mimiviridae, Herpesviridae, and Smacoviridae. *Lederbergvirus*, *Stanholtvirus*, *Punavirus*, and *Vieuvirus* were dominant in the virome at the genus level (Fig. 1D). When compared to control, the relative abundances of *Chlamydiamicrovirus* and *Cyclovirus* were lower in the Pb treatment (*p* < 0.05) (Supplementary Fig. 1).

The virome varied across all treatments (Adonis *R*² = 0.601, *p* = 0.001) (Supplementary Fig. 2C; Supplementary Table 4), yet the pairwise differences between treatments were not statistically significant. The differences in virome composition were associated with differences in APb content (adj. *R*² = 36.8%, *p* = 0.001) (Supplementary Table 5). Fitting all soil physicochemical variables using envfit revealed that pH and APb, AK, TN, and AN content were significantly associated with virome ordination (Supplementary Fig. 2D; Supplementary Table 5). Notably, the first two RDA axes explained a relatively small proportion of the total variance (RDA1 0.6%, RDA2 0.2%) for viral community ordination (Supplementary Fig. 2D), reflecting the high complexity of soil viromes and the limited explanatory power of measured soil physicochemical variables in present study.

### PLA and Pb restructure virus-host association

**Fig. 2:**
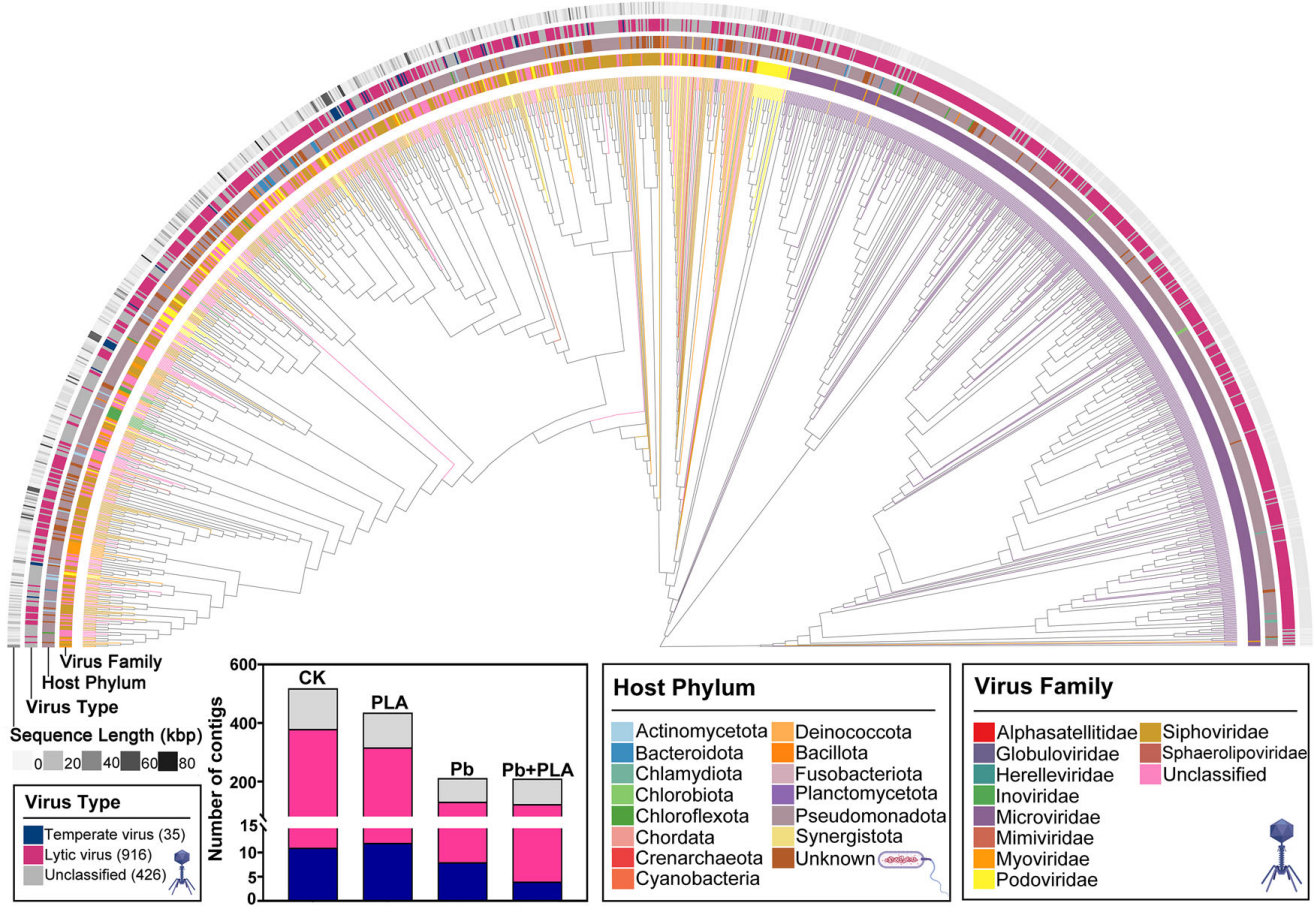
Predicted virus-host associations and lifestyle distribution (lytic vs. temperate virus) in buckwheat rhizosphere soil. Nodes represent bacterial phyla, viral contigs at the family level, and treatment replicates. Edge thickness corresponds to the number of linkages. CK, control with no PLA-MPs and Pb; PLA, polylactic acid microplastics treatment with 2 g PLA-MPs kg^−1^ soil; Pb, Pb treatment with 2 g Pb kg^−1^ soil; Pb+PLA, Combined Pb and PLA-MPs treatment with 2 g Pb and 2 g PLA-MPs kg^−1^ soil, *n* = 3 biologically independent samples.

Lytic viruses were predominant across all treatments, particularly in the CK and PLA treatments (369 and 305 contigs, respectively). In contrast, Pb and Pb+PLA treatments showed notably fewer lytic contigs (123 and 119, respectively). However, pairwise Fisher’s exact tests with FDR correction revealed no statistically significant differences in the number of lytic vs. temperate viruses among treatments (adjusted *p* > 0.05) (Fig. 2, Supplementary Table 7).

To investigate how contamination influenced virus-host associations, we analyzed viral contigs with predicted bacterial hosts (phylum level), focusing on phylum-family link patterns (Fig. 2; Supplementary Data 2). Pseudomonadota-Microviridae links were the most abundant across treatments, with 277, 38, 211, and 39 links in CK, Pb, PLA, and Pb+PLA, respectively. Following this, Siphoviridae was the second most common viral family associated with Pseudomonadota, with counts of 111, 37, 55, and 38 in the respective treatments. A total of 19 distinct phylum-family virus-host link types were observed in CK, 18 in Pb, 24 in PLA, and 20 in Pb+PLA. A Scheirer-Ray-Hare test using a reduced dataset (including link types with ≥5 observations) revealed a significant effect of Pb treatment on virus–host link composition (*H* = 24.88, df = 1, *p* < 0.05), while PLA treatment and interaction effects were non-significant. No significant differences were found in individual virus-host link types between treatments based on Fisher’s exact tests (FDR-adjusted *p* > 0.05), including links like Pseudomonadota-Siphoviridae and Actinomycetota-Siphoviridae (Supplementary Data 2). Notably, virus-host associations involving the Synergistetes were rare, with only one link in PLA. Most major clades corresponded to dominant virus families (e.g., Microviridae, Siphoviridae) and host phyla (e.g., Pseudomonadota, Actinomycetota).

### PLA and Pb drive divergent functional responses of rhizosphere bacterial and viral communities

**Fig. 3:**
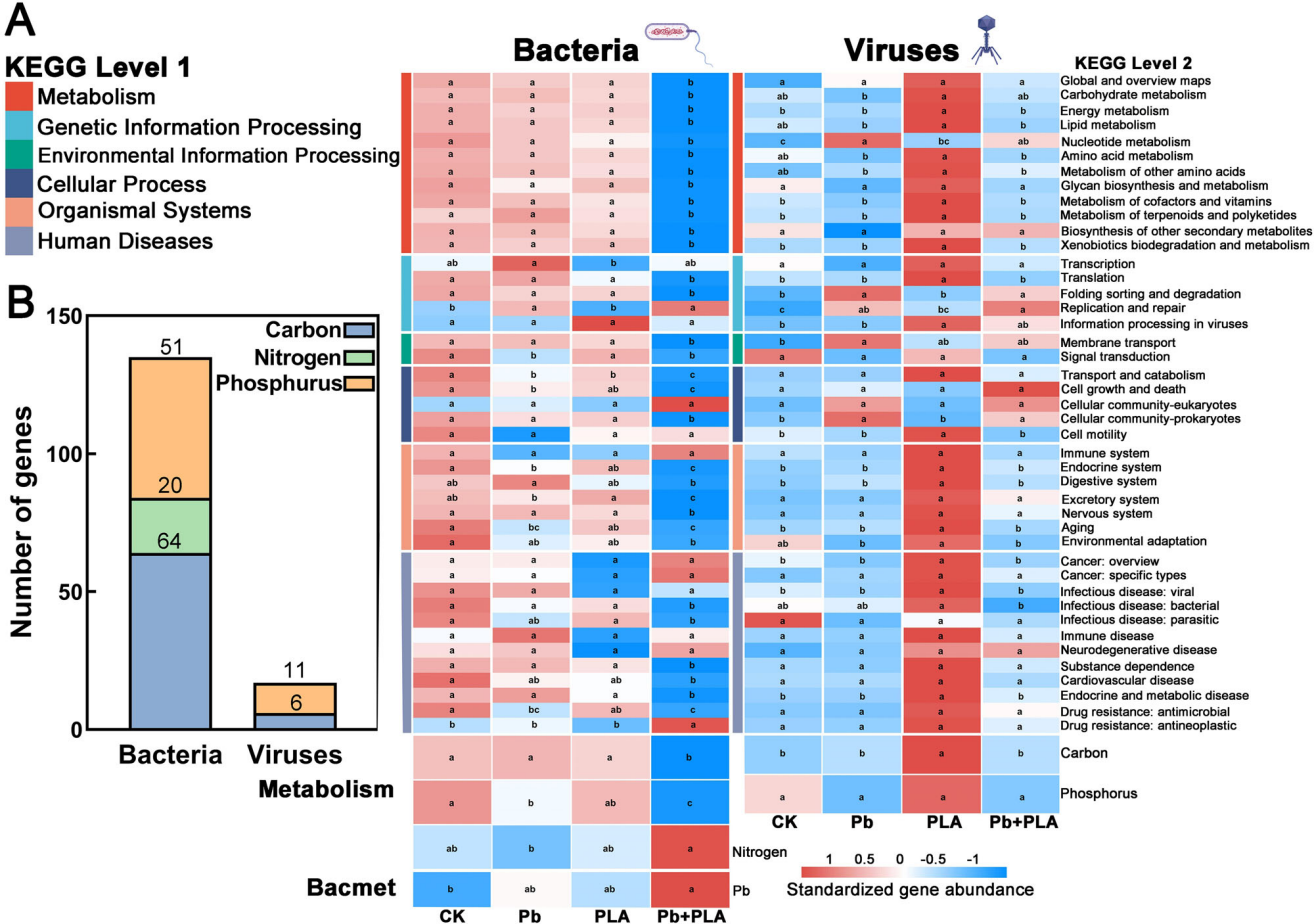
Functional annotation and relative abundances of bacterial and virus-encoded genes in buckwheat rhizosphere soil. **A** Relative abundances of functional genes annotated by KEGG and Bacmet databases, and genes linked to nutrient metabolism and Pb resistance genes in bacterial and viral metagenomes. **B** The number of bacteria and virus-encoded genes related to carbon, nitrogen, and phosphorus metabolism. CK, control with no PLA-MPs and Pb; PLA, polylactic acid microplastics treatment with 2 g PLA-MPs kg^−1^ soil; Pb, Pb treatment with 2 g Pb kg^−1^ soil; Pb+PLA, Combined Pb and PLA-MPs treatment with 2 g Pb and 2 g PLA-MPs kg^−1^ soil, *n* = 3 biologically independent samples.

Functional annotations of bacterial and viral communities under different treatments indicated variations in the profiles of functional genes, like KEGG level2 category, nutrient cycling-related genes, and Pb-tolerance related genes across the treatments (Fig. 3A, B; Supplementary Data 2). For bacterial community, a combination of Pb and PLA pollutants altered multiple functional categories such as carbohydrate metabolism, energy metabolism, amino acid metabolism, and xenobiotic biodegradation, which showed lower relative abundances in response to PLA and Pb treatment (*p* < 0.01) and strong PLA×Pb interaction effects (e.g., in energy metabolism, *p* < 0.001) (Supplementary Data 2). Carbon (C) and phosphorus (P) cycling genes were lower in abundance, while genes involved in nitrogen (N) cycling showed higher relative abundances under Pb+PLA pollution. The relative abundances of genes related to replication and repair were higher in the Pb-containing treatments than in the control (*p* < 0.01). Notably, genes related to Pb-tolerance were enriched in the bacterial community in the Pb+PLA treatment, and these genes were not detected in the viral community.

Regarding the viral community responses, the relative abundances of viral functional genes such as those involved in replication and repair and cell growth and death categories were higher under Pb and PLA treatments, especially under Pb+PLA (Fig. 3A) relative to the control. Furthermore, viral contributions to metabolic processes, particularly to carbohydrate metabolism was greater in the PLA-containing treatments relative to the control (Fig. 3A). Overall, the effects of the treatments on viral functions appeared smaller than those induced in bacterial communities.

**Fig. 4:**
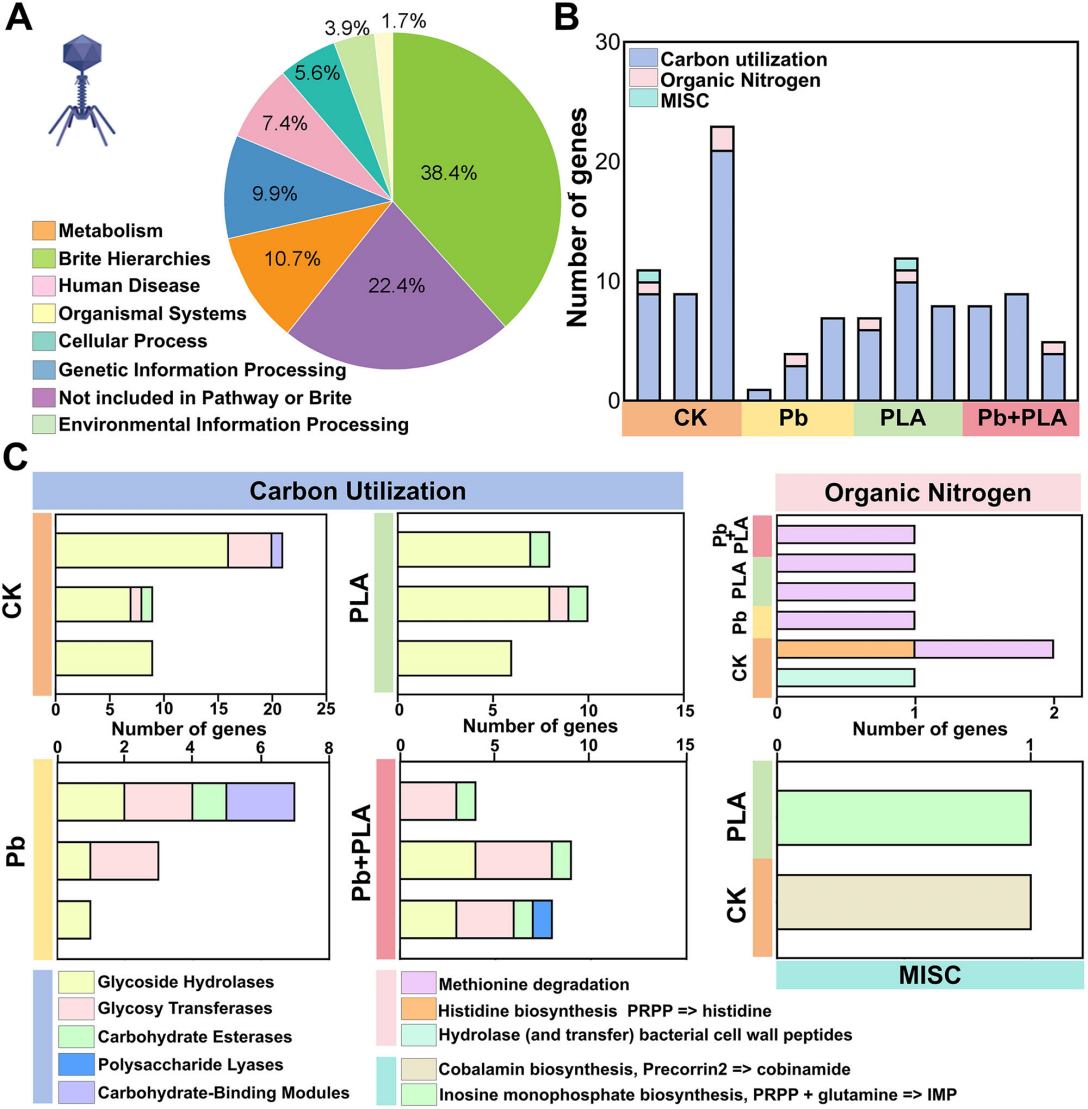
Functional annotation of virus-encoded auxiliary metabolic genes (AMGs) in buckwheat rhizosphere soil. **A** Relative distribution of virus-encoded AMGs across KEGG Level 1 categories. **B** The number of viral AMGs involved in carbon utilization, organic nitrogen metabolism, and miscellaneous (MISC) functions under each treatment. **C** Treatment-specific distribution of virus-encoded AMGs across functional sub-categories. CK, control with no PLA-MPs and Pb; PLA, polylactic acid microplastics treatment with 2 g PLA-MPs kg^−1^ soil; Pb, Pb treatment with 2 g Pb kg^−1^ soil; Pb+PLA, combined Pb and PLA-MPs treatment with 2 g Pb and 2 g PLA-MPs kg^−1^ soil, *n* = 3 biologically independent samples.

Analysis of virus-encoded AMGs across treatments showed that more than 38% of the annotated AMGs at KEGG level 1 were assigned to Brite hierarchies (Fig. 4A). Based on functional grouping of KEGG-annotated AMGs, viral genes were primarily assigned to categories related to carbon utilization, organic nitrogen metabolism, and miscellaneous metabolic functions (Fig. 4B). Functional genes, like glycosyl transferases, glycoside hydrolases, and carbohydrate esterases were identified in all treatments (Fig. 4C). Notably, carbohydrate esterases, including those with carboxylesterase-like activity, seemed more abundant in the Pb+PLA treatment, which is consistent with their known role in the hydrolysis of aliphatic polyesters such as PLA^24^. However, Fisher’s Exact Test showed no significant enrichment of any of the observed AMG categories across treatments (Supplementary Table 8). The viral AMGs were found to potentially carry key N cycle-related genes. Under the organic nitrogen category, methionine degradation was identified across treatments, while histidine biosynthesis, hydrolase, and transfer of bacterial cell wall peptides were only identified in the CK (Fig. 4C). The addition of PLA and Pb appeared to be associated with a decline in genes associated with organic nitrogen functionality in AMGs. Among the miscellaneous metabolic pathways, cobalamin biosynthesis was identified only in the control, whereas inosine monophosphate biosynthesis was detected in the PLA treatment.

### Putative PLA-microplastic degradation potential inferred from a virus-encoded AMGs

Comparison of PLA-associated enzyme commission (EC) numbers retrieved from the Plastics-Active Enzymes Database (PAZy) database with our annotated viral AMG dataset revealed carboxylesterase (CarE; EC 3.1.1.1) as a key candidate enzyme potentially involved in PLA degradation. A total of seven putative CarE genes were identified across viral contigs based on functional annotation and comparison with the PAZy database (Supplementary Fig. 3; Supplementary Table 9). Among these, five CarE genes were detected exclusively in PLA-containing treatments. The auxiliary scores of these CarE genes ranged from 1 to 3 (Supplementary Table 9). Notably, PLA3_contig_6091-cat_2_24 exhibited the highest auxiliary score, suggesting that although its viral origin was less certain, it remained a candidate for functional exploration. To select a representative gene for experimental validation, the candidate genes were cross-referenced with the PAZy database using National Center for Biotechnology Information (NCBI) BLAST accession numbers (e.g., BAC67195.1)^25^, followed by retrieval of corresponding KEGG orthology (KO) numbers. This process led to the selection of gene P9222_28545 (KO: K03930) (hereafter referred to as P92), which is annotated in KEGG as an esterase belonging to EC 3.1.1.-. Although P92 is currently annotated as a putative tributyrin esterase rather than a canonical CarE, PAZy annotations associate this enzyme with PLA degradation. Moreover, comparison with other esterase-related AMGs (e.g., EC 3.1.1.1 and EC 3.1.1.73; Supplementary Table 9) further supported the hypothesis that P92 may be involved in plastic degradation. Due to incomplete Open reading frames (ORFs) in viral CarE sequences, the P92 gene sequence was retrieved from the KEGG database and synthesized for experimental validation.

**Fig. 5:**
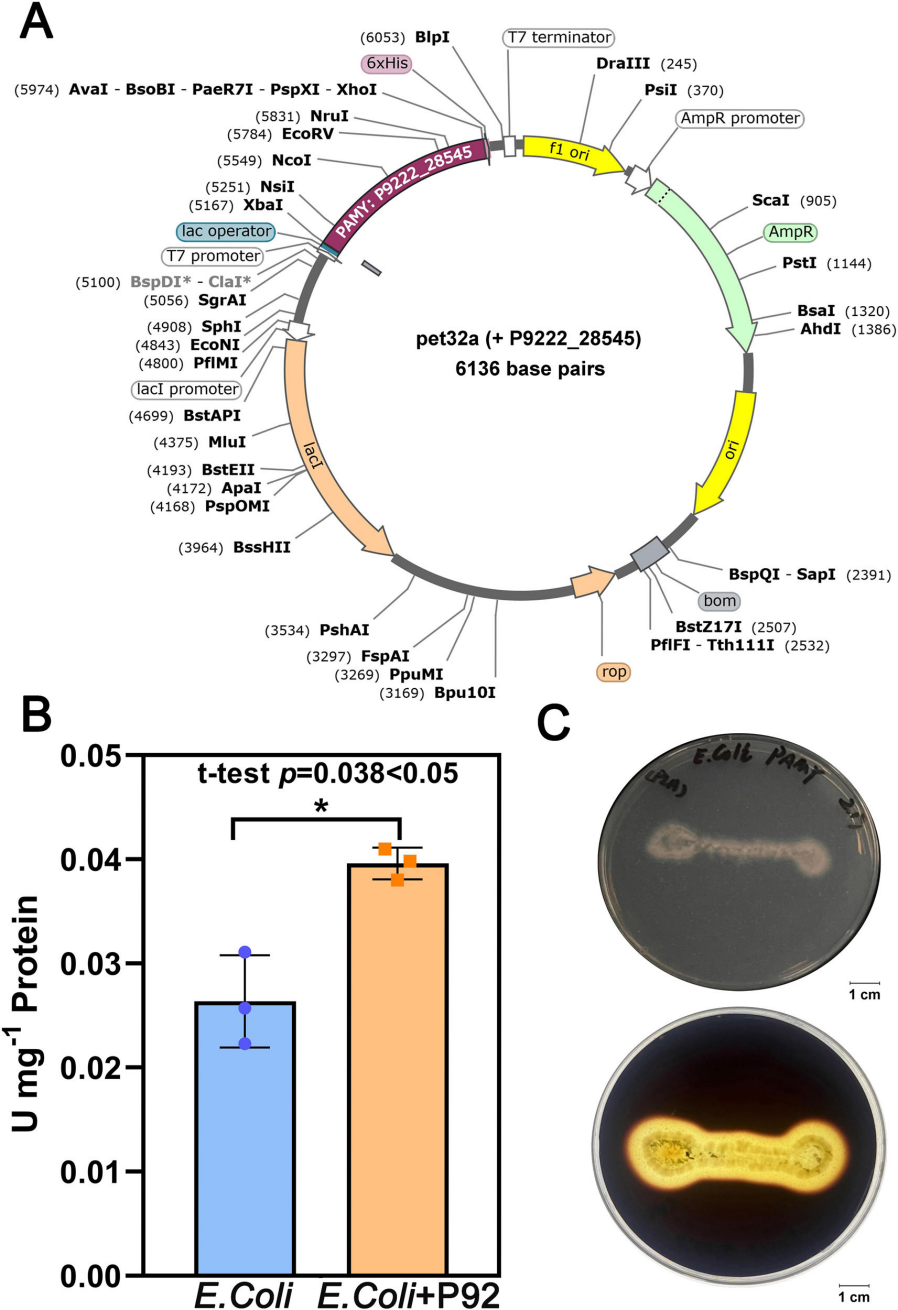
Functional validation of virus-encoded auxiliary metabolic genes (AMG) P9222_28545. **A** Schematic map of the recombinant expression vector pET-32a(+) harboring the gene P9222_28545. **B** Carboxylesterase activity of *E. coli* TOP10 (control) and recombinant strain *E. coli* TOP10 expressing P9222_28545 (*E. coli* + P92), *n* = 3 biologically independent samples. Error bars represent mean ± standard deviation (SD). **C** PLA degradation plate assay, the top plate: transparent degradation halo observed around *E. coli* + P92 on unstained PLA-containing medium; bottom plate: The halo became more distinct after potassium iodide (I_2_-KI) staining.

The gene P92 was subsequently cloned into the pET-32a (+) vector (*E*. *coli* + P92; Fig. 5A) and heterologously expressed in *E. coli*. Compared with the host strain (*E. coli* TOP10), the recombinant strain exhibited significantly higher esterase activity (0.039 ± 0.002 vs. 0.027 ± 0.004 U mg⁻¹ protein, *p* < 0.05; Fig. 5B), consistent with its annotation as a putative CarE. In addition, clear degradation zones were observed around colonies of the recombinant strain grown on PLA-containing medium (Fig. 5C), whereas no such halos were detected in the control strain (Supplementary Fig. 4).

## Discussion

We examined how PLA-MPs and Pb pollution applied individually or together influence the soil-buckwheat-microbiome system. Previous studies suggest that the negative effects of MPs on soil properties tend to be amplified in heavy metal-contaminated soils^5^. Our results showed that Pb addition lowered the soil pH, while PLA-MPs reduced SOC and increased Pb availability (Supplementary Table 1). This may be due to PLA-MPs decreasing soil adsorption capacity for metals and enhancing metal desorption^26^. PLA has also been reported to enhance cadmium (Cd) mobility by modifying soil conditions in a rice cultivation system^27^. Moreover, changes in SOC levels as shown in the present study were consistent with the reported impact of PLA-MPs on soil carbon cycling^7^.

Both PLA-MPs and Pb inhibited buckwheat growth, and a stronger inhibitory effect of Pb was observed (Supplementary Table 2). In related research, Liu et al.^27^ reported that PLA-MPs exhibited higher phytotoxicity than non-biodegradable MPs, resulting in lower rice shoot biomass. In our study, POD activity and plant Pb content were higher in the combined treatment than in the Pb treatment. Previous studies have suggested that MPs can influence the uptake and internal distribution of heavy metals in plants^28^. Consistent with this, we observed that both soil and plant shoot APb concentrations were slightly higher in the combined PLA-MPs and Pb treatment than in the Pb-only treatment. The significant higher POD activity in the combined Pb+PLA treatment indicates a heightened oxidative stress response in buckwheat due to the synergistic toxicit of these two pollutants. Although the activity of ALP was higher in the PLA-MPs treatment and lower in the Pb-containing treatment relative to the control, AP across treatments was similar (Supplementary Table 1); this indicates that the effects on plant growth were not P dependent. Furthermore, PLA-treated plants showed higher shoot fresh weight than Pb-treated plants, but their dry weight did not differ significantly, which may be attributed to differences in water retention or turgor, warranting further investigation.

The effects of PLA-MPs on soil bacterial diversity are dose-dependent, ranging from negative^4,7^ impacts to nonsignificant positive effects^29^. We found that although PLA-MPs or Pb alone had a limited impact on bacterial diversity, their combination reduced community diversity (Fig. 1A), indicating a synergistic stress effect. Changes in bacterial community composition were mainly attributed to Pb, with PLA playing a modulatory role by altering soil properties and Pb bioavailability. Notably, the combined treatment selectively enriched *Streptomyces, Rhodanobacter*, and *Ferruginibacter* (Supplementary Fig. 1), which are known for their heavy metal tolerance^30,31^, reported to proliferate in MP-contaminated soils^32^, and associated with PLA degradation^33^. These genera showed no significant changes under PLA-MPs alone but were enriched in both Pb and PLA combined treatments, suggesting that their proliferation was primarily driven by Pb stress, with the co-occurrence of PLA-MPs possibly enhancing this effect through altered soil properties or bioavailability of metals, as described above.

Although soil viromes remain underexplored in MPs-polluted soil, recent work by Wang et al.^34^ reported that bio-MPs treatments had lower viral diversity than the control. However, viral diversity remained largely unchanged in our study, likely due to the shorter exposure duration or moderate pollutant levels. Yet, in contrast, long-term or high-intensity metal contamination has been linked to both decreased^17,35^ and increased^36^ viral diversity, suggesting context-dependent virome responses to environmental stressors. Similar to the bacterial community, the Pb treatment induced pronounced shifts in viral community composition (Supplementary Fig. 2C; Supplementary Table 4). At the taxon level, the presence of PLA was associated with moderated Pb-related changes in viral composition. Specifically, the relative abundances of *Chlamydiamicrovirus* and *Cyclovirus* were reduced in the Pb treatment but remained stable in the PLA+Pb treatment (Supplementary Fig. 1), suggesting a counteractive effect of PLA on Pb-induced virome disruptions. This is reminiscent of findings in tea cropping systems, where soil pH changed the abundance of above virus genera^37^. Similarly, we found a strong correlation between soil pH and virome (Supplementary Table5), indicating that PLA may mediate viral responses indirectly via changes in soil chemistry. In addition, although direct evidence in soil systems remains limited, studies in aqueous environments have shown that viral particles can adsorb to MPs surfaces^38,39^, such interactions could potentially alter viral exposure to metal stress.

Viral lifestyle predictions further highlight distinct ecological dynamics: lytic phages dominated across all treatments, particularly under PLA and control conditions, whereas no increase in temperate phages was observed under Pb or PLA+Pb treatments (Fig. 2). This contrasts with previous reports that heavy metals and pesticides often induce shifts toward temperate lifestyles^18,36,40^, indicating that PLA-MPs alone did not exert a strong selective pressure for temperate viruses under present experimental conditions. Moreover, virus-host links exhibited strong Pb-associated variation (Supplementary Data 2). The total number of virus-host pairs declined substantially under Pb compared to the control, indicating suppressed viral propagation or reduced host availability. In contrast, PLA treatment retained higher levels of virus-host interactions, possibly by sustaining microbial activity or promoting the emergence of novel, unclassified viral lineages. Similar to findings in pesticide-contaminated soils, where Zheng et al.^40^ reported a greater number of bacterial taxa associated with viral contigs. We observed that virus-host connectivity persisted under PLA treatments, even without explicitly resolving viral host range. Consistent associations between major bacterial phyla (Pseudomonadota and Actinomycetota) and key viral families (Microviridae and Siphoviridae) were detected under treatments, indicating that short-term Pb or PLA exposure did not drive active host switching by viruses. These results corroborate the observation by Ji et al.^41^, where MPs triggered viral activation specifically within Pseudomonadota and Actinomycetota hosts. Furthermore, the high abundance of unclassified viral contigs in samples highlights the vast “viral dark matter” still hidden in terrestrial ecosystems^39^.

Distinct functional responses of bacterial and viral communities to Pb and PLA-MPs under the tested soil conditions. Overall, bacterial communities showed broader metabolic versatility compared to viral communities (Fig. 3A). This aligns with the ecological roles of bacteria as primary mediators of biogeochemical cycles, while viruses often contribute more subtly through AMGs^12,36^. Unlike the limited effects observed with PLA and Cd co-contamination on bacterial functional genes^29^, our results showed that Pb+PLA exposure led to a stronger suppression of microbial metabolic gene. While both studies suggest that bio-MPs can amplify metal-induced microbial responses, the synergistic toxicity was more evident under Pb stress. Specifically, Pb+PLA increased the abundance of bacterial genes involved in replication and repair, immune functions (Fig. 3A), and also altered viral AMG profiles (Fig. 4C), suggesting a stronger or more synergistic toxicity of Pb and PLA-MPs co-exposure. In contrast, viral communities exhibited more selective functional shifts. PLA alone enriched virus-encoded genes involved in C and P metabolism, while Pb+PLA co-treatment promoted genes related to replication and repair, indicative of heightened viral activity. Although direct lysis was not assessed, this pattern supports the “viral shunt” hypothesis^42^, in which viruses contribute to nutrient turnover both by reprogramming host metabolism through AMGs and by lysing host cells to liberate intracellular resources.

Bioplastic addition can alter microbial C, N, and P cycling in soil^43^. Here, bacterial genes involved in C and P metabolism were suppressed under Pb and PLA treatments, while N-cycling genes were enriched only under co-contamination. Previous reports have shown that Pb contamination can affect soil bacterial N cycling genes^44,45^. In our study, Pb likely altered nutrient dynamics or induced microbial stress responses that could have promoted N-cycling gene expression in bacteria, but not in viruses, although potential N cycle related AMGs were identified. These results suggest that bacterial communities respond more actively to PLA-MPs and Pb co-stress in terms of N metabolism, whereas viral involvement appears limited, possibly due to their dependency on host functions or constraints in viral genomic coding capacity. Although no KEGG-annotated N-cycle genes were detected, AMGs related to methionine degradation were present, while key functions like histidine biosynthesis appeared only in the control, suggesting pollutant-induced suppression of viral N-related functional potential^46^. Consistent with Huang et al.^47^, who linked viral AMGs to nutrient status, our results show that pollutant stress also reshapes viral functions, suppressing organic N genes while enhancing carbon degradation capacity. Soil conditions likely shaped these patterns: PLA-MPs maintained neutral pH and high ALP, favoring C/P-related viral functions, while Pb+PLA acidified the soil and reduced SOC, potentially limiting viral N cycling. Overall, bacteria responded more directly at the gene level, whereas viruses acted indirectly via AMGs. The divergent responses also suggest that bacterial and viral communities play complementary roles under stress, with bacteria driving core nutrient cycling and viruses modulating host-associated metabolism under selective pressures.

Despite growing recognition of viral AMGs in biogeochemical cycles, evidence of virus-mediated plastic degradation remains limited. While recent studies confirmed viral roles in degrading pesticides and chitosan^16,40^, the evidence of virus-encoded AMGs, particularly those involved in plastic degradation, remains scarce. Here, we observed an increase in carbon utilization-related AMGs under combined Pb+PLA treatments compared to individual Pb treatment. Among these, carbohydrate esterases, a class of enzymes known to hydrolyze ester bonds in biodegradable plastics, were enriched under PLA-addition (Supplementary Table 9), which suggests PLA may represent a selective pressure shaping virus-encoded AMGs in the rhizosphere. In such environments, viral AMGs are unlikely to function as independent degraders but may confer fitness benefits to hosts by modulating carbon utilization (Fig. 4C) and alleviating contaminant-associated stress. To assess the functional relevance of putative viral carboxylesterases, a candidate gene (P92) was heterologously expressed in *E coli*. The recombinant strain exhibited higher esterase activity compared with the host control and formed transparent halos on PLA-amended media, indicating enhanced hydrolytic activity toward ester-containing substrates. Together with the other results, this functional validation suggests that virus-encoded auxiliary genes that may help host to survive under PLA-polluted stress. It should be noted that the functional assays applied here reflect hydrolytic activity toward ester-containing substrates rather than complete PLA mineralization. Future studies should build on this framework to further explore the roles of viruses in the transformation or degradation of MPs contaminants in soil ecosystems.

## Methods

### Soil collection and pot experiment design

A pot experiment with four treatments, (i) control (CK, no pollutants), (ii) Pb (2 g Pb kg⁻¹ dry weight [DW] soil), (iii) PLA-MPs (2 g PLA kg⁻¹ DW soil), and (iv) Pb+PLA (2 g Pb + 2 g PLA kg⁻¹ DW soil) was carried out, each with three biological replicates, were conducted using buckwheat grown in agricultural soil from Huili, Sichuan, China (102°14′40.45″E, 26°39′18.97″N). Detailed soil characteristics and experimental conditions are provided in Supplementary Methods.

### Determining soil physicochemical and buckwheat properties

Soil physicochemical properties (i.e., pH, SOC, TN, AN, AP, AK), enzyme activities (i.e., ALP, ACP) were measured to characterize soil nutrients status and chemical condition potentially influenced by Pb and PLA^6^. For buckwheat, the macroscopic plant traits (i.e., height, root length, fresh and dry biomass, Pb accumulation) were determined to evaluate plant growth performance and Pb uptake. To assess oxidative stress, antioxidant responses, and photosynthetic status of buckwheat under Pb and PLA exposure, MDA content, antioxidant enzyme activities (i.e., POD, SOD), and chlorophyll content were measured by using standard protocols (see Supplementary Methods for details).

### Metagenomic sequencing and analysis of the bacterial fraction

DNA was extracted from soil samples using the FastDNA Spin Kit for Soil (MP Biomedicals, USA). Metagenomic libraries were constructed using the ALFA-SEQ DNA Library Prep Kit and sequenced on an Illumina platform (2×150 bp paired-end reads) by Guangdong Magigene Biotechnology Co., Ltd. (Guangzhou, China). Raw reads (average 9.60 × 10⁷ reads per sample; Supplementary Data 2) were quality-filtered using Fastp (v0.23.1)^48^ and Trimmomatic^49^. Clean reads were assembled using MEGAHIT^50^. Open reading frames (ORFs) were predicted using MetaGeneMark^51^ and Prodigal (v2.6.3)^52^, and clustered into a non-redundant gene catalog with Linclust^53^. Clean reads were mapped back to the gene catalog using BBMap^54^, and gene abundances were calculated based on read counts normalized by gene length. Functional annotation was performed by aligning predicted genes against the NCBI non-redundant (NR) protein database using DIAMOND^55^. Taxonomic assignment of bacterial genes was conducted using the lowest common ancestor (LCA) algorithm implemented in MEGAN 4^56^, based on BLASTn searches against the NCBI nucleotide (NT) database (*E* ≤ 10⁻⁵). Relative abundances of bacterial taxa were calculated as the proportion of taxonomically assigned reads per sample and used to characterize community composition. Phylum names were reported using ICNP-valid nomenclature (suffix “-ota”) following Oren (2024), LPSN, and NCBI taxonomy standards^57^. Detailed parameters for quality filtering, assembly, gene prediction, and annotation are provided in the Supplementary Methods.

### DNA extraction and sequencing of viral fraction

Viruses were collected using the ultracentrifugation method as previously described by Szpara^58^; detailed method is provided in Supplementary Methods. The resulting viral suspension was then used for nucleic acid extraction. Viral nucleic acids were extracted using a TaKaRa MiniBEST Viral DNA Extraction Kit Ver.5.0 (Takara Bio Inc., Shiga, Japan), followed by whole-genome amplification using an Illustra Ready-To-Go GenomiPhi V3 DNA Amplification Kit (Cytiva, Marlborough, USA). Amplified products were quality-checked using Thermo NanoDrop One (Thermo Fisher Scientific, MA, USA), Qubit 4.0 Fluorometer (Life Technologies, USA), and 1.5% agarose gel electrophoresis. Viral metagenomic libraries were constructed using the ALFA-SEQ DNA Library Prep Kit (mCHIP, Guangzhou, China), and paired-end sequencing (2×150 bp) was performed on the Illumina NovaSeq 6000 platform at Guangdong Magigene Biotechnology Co., Ltd. (Guangzhou, China), following the manufacturer’s protocol. Raw viral reads were quality-filtered using Trimmomatic (v. 0.40)^49^ to remove low-quality bases and adapter sequences. To minimize host-derived contamination, quality-filtered reads were screened against a custom microbial host genome database using BWA (v. 0.7.17)^59^, followed by de novo assembly with MEGAHIT (v.1.2.9)^50^. Assembled contigs were further checked against the same host database using BLASTn to remove residual host sequences, and clean reads were mapped back to contigs to evaluate assembly efficiency (detailed methods are provided in Supplementary Methods).

### Identification and classification annotation of viral sequences

Putative viral contigs were identified using a combined pipeline integrating CheckV (v0.8.1)^22^ and VirSorter2^23^ (v2.2.3). High- and medium-quality viral contigs from both tools were retained and dereplicated using CD-HIT (v. 4.8.1)^60^. Taxonomic classification was performed with PhaGCN2 (v. 2.0)^61^ and cross-validated against the latest ICTV Master Species List, while traditional morphotype-based family names were retained for consistency with existing viromics literature^62^. Detailed methods are in Supplementary Methods.

### Viral abundance statistics

Viral reads were aligned to the identified viral contigs, and Reads Per Kilobase per Million mapped reads (RPKM) values were calculated for each contig using the equation^63^:

$${{{\rm{RPKM}}}}=\frac{{{{\rm{Contig}}}}\; {{{\rm{reads}}}}}{{{{\rm{Total}}}}\; {{{\rm{mapped}}}}\; {{{\rm{reads}}}}\left({{{\rm{millions}}}}\right)* {{{\rm{Contig}}}}\; {{{\rm{length}}}}({{{\rm{KB}}}})}\,$$

where contig reads are the number of reads mapped to a specific contig, total mapped reads are the total number of reads that have been mapped, expressed in millions, and contig length is the length of a contig in kilobases (KB). For viral community composition analyses, relative abundances at the taxon levels were calculated by summing RPKM-normalized contig abundances assigned to each taxon and expressing them as proportions of the total viral RPKM per sample.

### Host predictions, lifestyle predictions, and auxiliary metabolic gene (AMG) annotation for viral fraction

Hosts of viral contigs were predicted using CHERRY (v. 2023)^64^ and PHP (v. 2023)^65^, followed by threshold-based filtering to retain high-confidence host assignments (PHP score ≥ 1400; recall = 91.6%, precision = 99.1%). Only viral contigs ≥1 kb with reliable bacterial host annotations were retained for downstream analyses. Viral phylogenies were annotated with lifestyle, contig length, viral family, and host phylum to characterize host-linked viral traits. To assess contamination-driven shifts in virus–host association structure, host–virus links were summarized at the phylum–family level and compared across treatments, excluding low-confidence predictions involving non-bacterial hosts or single-occurrence links.

Temperate viruses were conservatively confirmed by screening for prophage signals, identified using VIBRANT v1.2.1^66^ and manual detection of temperate-specific genes. The remaining contigs that displayed no prophage signals or temperate-specific genes were considered as potential lytic viruses.

Viral genes were predicted, type-annotated, and classified using Virsorter2^23^. Genes within virus sequences were scored based on their respective categories using DRAM-v (v.1.2.0)^67^. Sequences with a virus prediction score >0.5 and a length >5 kb were selected for further analysis. Functional annotation was performed using an integrated multi-database approach. Predicted protein sequences were queried against Pfam (http://pfam-legacy.xfam.org/)^25^, UniProt (https://www.uniprot.org/)^68^, MEROPS^69^, CAZy (www.cazy.org/)^70^, and the Virus Orthologous Groups database (VOGDB) (VOGDB: vog203, http://vogdb.org/)^71^ using sequence similarity- and profile-based searches. Annotations from different databases were integrated to assign functional categories. When multiple annotations were available for a given gene, the best-supported annotation based on database specificity and confidence was retained. Based on these integrated annotations, the putative types of viral AMGs were designated.

### Bacterial and viral gene annotation

Further functional annotation of bacterial and viral genes was performed using DIAMOND (v. 0.9.14)^55^ with an *e*-value cutoff of 10^-5^. Functional genes linked to nitrogen, carbon, and phosphorus cycling were identified based on metabolic pathways mapped to viral KO terms in the KEGG database^72^. Bacterial and viral functional genes were summarized at KEGG level 2 to enable comparison of specific biogeochemical processes. Given the relatively limited number and uneven functional distribution of virus-encoded AMGs, functional annotation of them was summarized at KEGG level 1. Bacterial Pb resistance genes were identified using the Antibacterial Biocide & Metal Resistance Gene (BacMet) Database^73^. The relative abundances of genes were calculated by summing the values assigned to each functional category, as defined in the KEGG or BacMet annotations.

### Screening of putative PLA-degrading enzymes

To identify potential PLA-degrading enzymes from virus-encoded AMGs, we performed functional annotation comparison between our viral AMGs dataset and the PAZy (https://pazy.eu/doku.php?id=start), which catalogs experimentally verified plastic-degrading enzymes. EC numbers associated with PLA degradation were retrieved from the PAZy database and cross-referenced with our annotated AMG dataset to identify candidate AMGs encoding potential PLA-degrading enzymes. Candidate AMGs were further classified based on their EC annotations, auxiliary scores, and AMG flags.

### Experimental validation of putative PLA-degrading enzymes

Selected candidate genes were subjected to experimental validation through heterologous expression and enzymatic assays. Briefly, candidate genes were synthesized and cloned into the pET-32a ^(+)^ expression vector and transformed into *Escherichia coli* (*E*· *coli*). Recombinant strains were screened on LB agar plates supplemented with ampicillin (50 μg mL⁻¹) and verified by colony PCR and sequencing. To assess potential PLA degradation, recombinant strains were inoculated onto PLA-containing mineral salt medium with emulsified PLA as the sole carbon source (see Supplementary Methods for detailed composition), where the formation of transparent halos around colonies was used as an indicator of PLA hydrolysis. Iodine–potassium iodide (I₂–KI) staining was applied to enhance halo visualization by staining residual polymeric PLA dispersed in the agar matrix. Areas where PLA polymers were depleted due to enzymatic hydrolysis remained unstained, resulting in clear zones around colonies^74^. In addition, esterase activity was quantified using a commercial esterase assay kit (Colorimetric Method, Sangon Biotech, CHN), with corresponding host strains serving as controls (see Supplementary Methods for details).

### Statistics and reproducibility

Statistical tests and visualization were performed using GraphPad Prism 8.0 (https://www.graphpad.com/), IBM SPSS 26 (https://www.ibm.com/spss) and R v.3.6.2 in R Studio v. 4.4.3 (https://www.r-project.org/). Differences were considered statistically significant at *p* < 0.05. Experiments were conducted with three biologically independent replicates per treatment, each representing an independent soil sample (pot). Experiments without formal statistical analysis, such as the plate-based degradation assay, were independently repeated at least three times with consistent results. Before analysis, all datasets were tested for normality using the Shapiro–Wilk test and for homogeneity of variance using Levene’s test. For datasets meeting parametric assumptions, differences in soil properties, alpha diversity indices, and functional gene abundances were assessed using two-way ANOVA followed by Tukey’s multiple range test in SPSS 26.0. Differences in viral lifestyle and AMG functional categories were tested using Fisher’s exact test; multiple *P*-values comparison was implemented using the Benjamini–Hochberg false discovery rate (FDR) method. Differences in host phylum–virus family link composition were first assessed using the Scheirer–Ray–Hare test implemented in the R package rcompanion^75^ to evaluate overall shifts in virus–host association structure across treatments, followed by Fisher’s exact tests to examine treatment effects on individual phylum–family links. Figures were generated with R packages ggplot2 v.3.4.4, ggpubr v.0.6.0, and ggrepel v.0.9.4^76,77^. Differences in beta-diversity were visualized using principal coordinates analysis (PCoA) based on the Bray-Curtis distance matrix, and tested using permutational multivariate analysis of variance (PERMANOVA) and pairwise PERMANOVA with 999 permutations in R packages vegan v2.6.4 and pairwiseAdonis v. 0.4.1^78,79^. Differential abundance analysis at genus level was performed using the ancombc2 function in R package ANCOM-BC (v.2.0.2)^80^. Twenty genera with highest relative abundances across all samples were included in the analysis. Phylogenetic trees were constructed using VIPtreeGen, and visualized with R packages ggtree, ggtreeExtra, ggplot2, ggnewscale, and treeio^81^.

To assess the relationship between the bacterial and viral community composition and environmental variables, redundancy analysis (RDA) was performed using the vegan package (8.0) in R. Environmental predictors of community composition were selected using forward selection based on adjusted R² and permutation tests (*n* = 999). Associations between ordination axes and environmental variables were further evaluated using the envfit function in vegan package.

### Reporting summary

Further information on research design is available in the Nature Portfolio Reporting Summary linked to this article.

## Supplementary information

Transparent Peer Review file

Supplementary Information

Description of Additional Supplementary files

Supplementary Data1

Supplementary Data2

reporting summary

## Data Availability

The raw bacterial and viral metagenomic sequencing data generated in this study have been deposited in the NCBI Sequence Read Archive (SRA) under BioProject accession numbers PRJNA1266835 and PRJNA1266889. All datasets are publicly accessible via the NCBI BioProject database. Source data underlying the figures are provided as Supplementary Data 1. All other data supporting the findings of this study are available from the corresponding author upon reasonable request.
