# Supplementary material for: Microplastic and lead shift microbiomes enriching viral auxiliary metabolic genes for potential polylactic acid degradation
Source: Commun Biol. 2026 May 7;9:949. doi: 10.1038/s42003-026-10162-7 (PMC13365211; doi:10.1038/s42003-026-10162-7)
Supplement: Supplementary file 2 — Supplementary Information [file 42003_2026_10162_MOESM2_ESM.pdf]

Supplementary Information

Microplastic and Lead Shift Microbiomes, Enrich Viral Auxiliary Metabolic Genes for Potential Polylactic Acid Degradation

Xieluyao Wei[1†], Kinza Bashir[1†], Xianrui Tian[1†], Akasha Farooq[1†], Expedito Olimi[2,3], Tomislav Cernava[2], Lingzi Zhang[1], Xiumei Yu[1], Qiang Chen[1], Petri Penttinen[1]*, Yunfu Gu[1]*

1. College of Resources, Sichuan Agricultural University, Chengdu, China

2.School of Biological Sciences, Faculty of Environmental and Life Sciences, University of Southampton, Southampton, United Kingdom

3. Institute of Environmental Biotechnology, Graz University of Technology, Graz, Austria

†: These authors contributed equally

[*] Corresponding authors

E-mail address: petri.penttinen@helsinki.fi

guyf@sicau.edu.cn

This supplementary information includes:

Supplementary Methods

Supplementary Figure 1-4

Supplementary Table 1-9

Supplementary Methods

Soil Collection and Pot Experiment Design

Soil samples were collected from the topsoil layer (0–20 cm) of an actively cultivated agricultural field in Huili, Sichuan, China. Prior to sampling, visible plant residues, leaf litter, and larger stones were removed from the soil surface as a standard pre-treatment to ensure homogeneity and to avoid interference from undecomposed organic debris. Baseline analyses of the soil physicochemical properties, including pH and soil organic matter (SOM) content, soil organic carbon (SOC), total nitrogen (TN), alkaline hydrolysable nitrogen (AN), available phosphorus (AP), and available potassium (AK) were tested using the methodology described by Zhang and Gong[1]. The total Pb and available Pb (APb) content of the soil were tested according to the method described by Feng et al.[2]. The basic soil properties of collected soil were as followed: pH 6.55±0.04; SOC 10.11±0.32 g kg^{-1}; TN 1.10±0.02 g kg^{-1}; AN 43.36±0.48 mg kg^{-1}; AP 30.89±0.59 mg kg^{-1}; AK 301.60±1.63 mg kg^{-1}; Total Pb 33.36±0.28 mg kg^{-1}; APb 0.27±0.01 mg kg^{-1}. Based on the '*Risk control standard for soil contamination of agricultural land*' (China's soil environmental quality for agricultural land standard, GB 15618–2018), the total Pb concentration of collected soil was far lower than the 'risk screening value' (33.36 mg kg^{-1} vs 120 mg kg^{-1}). Therefore, the soil used in this study can be considered uncontaminated with Pb.

The pot experiment was designed to evaluate how lead (Pb) and PLA-MPs, alone and in combination, affect the taxonomic and functional composition of buckwheat rhizosphere bacterial and viral communities. The experiment included four treatments: (i) control (CK, no pollutants), (ii) Pb (2 g Pb kg^{-1} soil), (iii) PLA-MPs (2 g PLA kg^{-1} soil), and (iv) Pb+PLA (2 g Pb + 2 g PLA kg^{-1} soil) (based on dry soil), each with three biological replicates. The concentrations of PLA and Pb were selected to represent an elevated exposure scenario rather than average background levels in agricultural soils. For MPs, reported concentrations in agricultural soils vary widely and can reach to 2000 items kg^{-1} soil in intensively managed systems receiving long-term plastic mulching, sludge amendment, or organic fertilizers[3]. Similarly, the applied Pb concentration was chosen to induce measurable biological responses without exceeding phytotoxic thresholds[4].

Lead treatments were prepared by dissolving $Pb(NO_3)_2$ in distilled water and evenly spiking the soil to achieve the target Pb concentration. The PLA microplastics used in this study had particle sizes of 13–15 μm and a reported polymer purity of 99%, and were obtained from Dongguan

Huachuang Plastic Chemical Co., Ltd. (Dongguan, Guangdong, China)., specific treatment protocols were based on our previous study[5]. Seeds of buckwheat (*Fagopyrum tataricum* L. Gaertn.) were germinated at 25°C for 48 h in Petri dishes, then transplanted into pots (containing 2.5 kg of dry soil). The pots were maintained at 25°C and 80% humidity in a conditioned greenhouse of the Biotechnology Laboratory of the Sichuan Agricultural University, Chengdu, China. The soil moisture was maintained at 60% of the soil's maximal water-holding capacity. The experiment was maintained for 60 days, and the plants were harvested to obtain soil for the analysis. After shaking each root gently, we carefully collected the soil adhering to the roots with forceps and defined it as rhizosphere soil[6]. Moreover, the soil that did not adhere to the fine roots was labelled bulk soil. The rhizosphere soil was stored at -80°C for metagenomic analyses (bacteria and viruses). Bulk soil samples were divided into two portions: one stored at 4°C to determine soil enzyme activities, and the remaining portion air-dried for the analysis of physicochemical properties and Pb content.

Determining Soil Physicochemical and Buckwheat Properties

Soil samples were ground and sieved using a 2 mm mesh, and soil physico-chemical analyses, like examining AN, SOC, TN, AK, AP, and APb content. The activities of alkaline phosphatase (ALP) and acid phosphatase (ACP) enzymes were measured as described previously[5]. Buckwheat plant traits were recorded at harvest, including plant height, root length, fresh and dry biomass. In addition, oxidative stress biomarkers, including malondialdehyde (MDA) content, the activities of superoxide dismutase (SOD), peroxidase (POD) and the leaf chlorophyll were measured in fresh leaf samples according to the method described by Chen et al[7].

For the APb determine, the dry soil and buckwheat samples (1 g) were digested with 5 mL of concentrated HNO_3 and aqua regia (15 mL, 70% Conc. HNO_3 and 65% $HClO_4$; 2:1) and heated until white fumes appeared at 80°C. After filtering, the digested mixture was diluted with purified water to make a final amount of 50 mL, and then using a calibrated atomic absorption spectrophotometer to test the lead level in samples[8].

Metagenomic Sequencing and Analysis of the Bacterial Fraction (detailed procedures)

Microbial DNA was extracted using the FastDNA Spin Kit for Soil (MP Biomedicals, Irvine, USA). Qualified DNA samples were then subjected to library construction using the ALFA-SEQ

DNA Library Prep Kit, wherein samples were subjected to DNA fragmentation, end repair, adapter ligation, PCR amplification, and purification. The quality and size distribution of the libraries were assessed using the Qsep400 system and Qubit 4.0. Metagenomic libraries were sequenced on the Illumina platform with 2×150 bp paired-end reads by Guangdong Magigene Biotechnology Co., Ltd. (Guangzhou, China). Raw sequence data, with an average read count of 9.60×10^7 reads per sample (Supplementary Dataset 1), were quality-filtered using Fastp (v. 0.23.1)[9] with parameters: -5 -W 5 -M 20 -q 15 -u 40 -l 50 –dedup. Low-quality reads were further filtered using the Trimmomatic toolkit[10]. The clean reads were assembled into scaftigs using MEGAHIT[11]. Open reading frames (ORFs) were predicted using MetaGeneMark v.3.38[12] and further clustered with Linclust[13]. ORF prediction and annotation were performed on scaftigs (⩾500 bp) from individual samples and mixed assemblies using Prodigal (v.2.6.3)[14]. Cleaned reads were mapped back to the non-redundant gene catalog using BBMap (http://jgi.doe.gov/data-and-tools/bbtools/) to obtain read counts per gene[15]. Gene abundances were calculated based on mapped read counts normalized by gene length. Relative abundance of bacterial taxa was calculated as the proportion of reads assigned to each taxon relative to the total number of taxonomically assigned reads per sample, and was used to describe compositional shifts in bacterial community structure. Functional annotation was performed by aligning predicted genes against the NCBI non-redundant protein database (NR) using DIAMOND[16]. Taxonomic assignment of bacterial genes was conducted using the lowest common ancestor (LCA) algorithm implemented in MEGAN 5[17], based on BLASTn searches against the NCBI nucleotide database (NT) (E-value$\leqslant 10^{-5}$). Based on LCA results, gene average depths and abundances were summarized at different taxonomic levels (from kingdom to species).

Viruses Collected from Soil Samples

Briefly, 2 g of a soil sample was ground and added to five volumes of pre-cooled sterile Stabilization Buffer (SB). The mixture was vortexed for 5 mins, subjected to three rounds of freezing-thawing in liquid nitrogen, and then centrifuged at 12000 ×g for 5 mins. Cellular debris in the supernatant was eliminated by filtration through a dual-layer membrane filter with pore sizes ranging between 0.45 μm and 0.22 μm. The supernatant was transferred into an ultracentrifuge tube containing 28% (w/w) sucrose and centrifuged at 160000 ×g for 2 h at 4°C using a Himac CP 100WX ultracentrifuge (Hitachi, Tokyo, Japan). After removing the supernatant, the pellet was

resuspended in 200 μL of SB. Subsequently, 8 μL of Enzyme Mix Buffer (EMB) and 2 μL of Enzyme Mix (EM) were added to the suspension (i.e., in a ratio of 4:1:20 for EMB: EM: SB, as recommended by the manufacturer). The mixture was incubated at 37°C for 60 min. Then, 2 μL of Stop Solution (SS) was added to terminate the reaction, followed by inactivation of enzymatic activity at 65-75°C for 10 min. After centrifugation at 2000 rpm for 5 minutes, 200 μL of the supernatant was collected and stored at -20°C for subsequent analyses[18].

Removal of Host Contamination and Assembly Pipeline

To reduce host-derived contamination, high-quality reads were mapped against a custom microbial host database (including representative bacterial, archaeal, and fungal genomes) using BWA (v. 0.7.17)[19] with the mem-k30 setting. Reads with alignment lengths less than 80% of their total read length were discarded. The remaining clean reads were then *de novo* assembled using MEGAHIT[11] (v.1.2.9) with the parameters --presets meta-large --min-contig-len 300. The assembled contigs were subsequently aligned to the same host genome database using BLASTn (v.2.9.0+) to remove residual host sequences. Mapping of the quality-filtered reads back to assembled contigs was performed using BWA to assess assembly efficiency and read utilization.

Identification and Classification Annotation of Viral Sequences

Potential viral sequence sets within the assembled sequences were predicted using the CheckV software (v. 0.8.1)[21]. Briefly, host contamination was identified via a hidden Markov model (HMM), and prophages were predicted. Potential viral sequences were discerned, and their confidence and completeness assessed through amino acid identity (AAI) comparisons and HMM-based identification. CheckV was used to further evaluate whether the viral sequences with high completeness (>90%) possessed direct terminal repeats (DTRs) or inverted terminal repeats (ITRs) at both ends, thereby predicting the presence of a complete viral structure. Lastly, candidate viral sequences underwent quality assessment based on their sequence completeness and direct/inverted terminal repeat (DTR/ITR) features. In parallel, the assembled contigs were further screened using VirSorter2 (v2.2.3)[21], which identifies viral sequences based on gene content and genomic structural features. High-confidence viral sequences were first defined based on CheckV quality assessment (i.e., high- and medium-quality viral contigs). VirSorter2 results were then used as a complementary

screening step to recover additional putative viral contigs that were not retained by CheckV but showed strong viral signatures. By integrating CheckV-based quality classification with VirSorter2 predictions, we enhanced the sensitivity of viral detection while retaining stringent confidence criteria for downstream analyses.

Candidate viral sequences identified in each sample were subjected to clustering and redundancy removal using the PSI-CD-HIT script within CD-HIT (v. 4.8.1)[22,23]. Taxonomic annotation of putative viral sequence sets was performed using PhaGCN2 (v. 2.0), a semi-supervised machine learning model based on graph convolutional neural networks, which can rapidly infer the most recent viral taxonomy standards issued by the International Committee on Taxonomy of Viruses (ICTV) (https://ictv.global/). The species information of the viral sequences was jointly confirmed by integrating the annotation results from PhaGCN2 with the annotated information of target alignment sequences in databases. Ultimately, the viral sequences were classified according to the viral identification method, confidence level, and completeness information. Viral taxon names were subsequently cross-checked against the latest ICTV Master Species List (MSL #40, v2)[24]. Traditional morphotype-based family names (e.g., Podoviridae, Myoviridae, Siphoviridae) were retained for consistency with current viromics pipelines and comparative literature.

PLA Degradation Assay and Carboxylesterase Activity Measurement

After overnight activation in LB medium (Peptone 10 g L^{-1}, yeast extract 5 g L^{-1}, NaCl 5 g L^{-1}, pH=7.0~7.2), strains (*E. coli* harboring pET-32a $^{(+)}$ with the target gene) were streaked onto PLA-selective media (the modified mineral salt medium (MSM) used for PLA selection contained emulsified PLA (1.00 g L^{-1}) as the sole carbon source, $(NH_4)_2SO_4$ 1.00 g L^{-1}, NaCl 0.02 g L^{-1}, $CaCl_2$ 0.05 g L^{-1}, $MgSO_4 \cdot 7H_2O$ 0.10 g L^{-1}, $FeSO_4 \cdot 2H_2O$ 0.02 g L^{-1}, K_2HPO_4 1.60 g L^{-1}, and KH_2PO_4 0.20 g L^{-1}. For solid media preparation, agar was added at 15.00 g L^{-1}, and the pH was adjusted to 7.0~7.2. The plates were incubated at 37°C for 48 h and subsequently stained with an iodine–potassium iodide (I_2–KI) solution. The formation of clear halos around colonies was taken as an indication that the microorganism was able to utilize PLA as carbon source, suggesting a potential capacity for PLA degradation[25,26].The emulsification of PLA was performed to improve the dispersion and homogeneity of this hydrophobic polymer in aqueous culture media, thereby increasing its effective surface area and ensuring reproducible microbial access to the substrate, as described in previous

studies, with corresponding host strains (*E· coli*) serving as the control.

When testing carboxylesterase activity, the recombinant strains (*E. coli* harboring pET-32a (+) with the target gene) were cultured in PLA-selective liquid medium supplemented with 1-naphthyl acetate. 1-Naphthyl acetate is a widely used surrogate substrate for carboxylesterases, enabling sensitive and quantitative detection of ester bond–hydrolyzing activity[27]. After 48 h of incubation at 37°C, 2 mL of bacterial culture was harvested by centrifugation at 4000 rpm for 10 min. The supernatant was used directly for enzyme activity determination. Carboxylesterase activity was measured using a commercial colorimetric Carboxylesterase (CarE) Activity Assay Kit (Carboxylesterase Activity Assay Kit, Colorimetric Method, Sangon Biotech, CHN), following the manufacturer's instructions. Briefly, the assay is based on the hydrolysis of 1-naphthyl acetate to 1-naphthol by carboxylesterase. The released 1-naphthol subsequently reacts with Fast Blue salt to form a stable azo compound, which exhibits a characteristic absorbance at 450 nm. Reaction mixtures were incubated at 37°C, and absorbance was recorded at defined time points using a visible spectrophotometer. Enzyme activity was calculated based on the change in absorbance (ΔA) according to the manufacturer's formula and normalized to protein content. Corresponding host strains (*E· coli*) were included as controls. However, we explicitly acknowledge the limitations of present approaches. The assays employed here do not directly quantify PLA mineralization, nor do they assess PLA-derived monomers, oligomer size distributions, changes in molecular weight, or CO_2/CH_4 production. Therefore, the observed halo formation and increased esterase activity are interpreted as evidence for potential involvement in the initial hydrolytic or depolymerization steps of PLA.

Supplementary Table 1 Soil properties in the treatments

Treatments	pH	Alkaline nitrogen (mg kg^{-1})	Soil organic carbon (g kg^{-1})	Available Pb (mg kg^{-1})	ALP (nmol h^{-1} g^{-1})
CK	6.19±0.07a	78.55±9.06bc	10.14±0.60a	2.21±0.10c	71.47±24.95b
Pb	5.58±0.05b	92.53±7.20ab	8.98±0.61ab	945.12±12.34b	28.78±4.01c
PLA	6.28±0.04a	50.6±4.01c	8.47±0.78b	2.26±0.06c	140.89±2.14a
Pb+PLA	5.71±0.03b	104.57±13.75a	9.16±0.22ab	991.78±14.09a	32.18±3.21c

Treatments	ACP (nmol h^{-1} g^{-1})	Total nitrogen (g kg^{-1})	Available potassium (mg kg^{-1})	Available phosphate (mg kg^{-1})
CK	1134.00±60.16a	1.18±0.03a	318.03±7.89a	60.22±5.10a
Pb	1146.29±45.15a	1.29±0.14a	371.20±34.19a	43.79±2.99a
PLA	1324.03±51.25a	1.12±0.08a	321.71±14.11a	61.01±2.12a
Pb+PLA	1236.90±78.12a	1.25±0.02a	350.06±20.34a	44.82±3.75a

Data are mean±SD (n=3). Different superscript letters in a column indicate statistically significant differences (Tukey's multiple range test, $p< 0.05$). ALP, alkaline phosphatase; ACP, acid phosphatase. CK, control with no PLA-MPs and Pb; PLA, polylactic acid microplastics treatment with 2 g PLA-MPs kg^{-1} soil; Pb, Pb treatment with 2 g Pb kg^{-1} soil; Pb+PLA, Combined Pb and PLA-MPs treatment with 2 g Pb and 2 g PLA-MPs kg^{-1} soil.

Supplementary Table 2 Effects of PLA, Pb, and their interactions with soil and buckwheat properties based on a two-way ANOVA analysis.

Sample		Parameter	PLA	Pb	PLA × Pb
Soil		pH	2.098	49.124***	0.561
		Total nitrogen	1.355	5.918*	0.179
		Alkaline nitrogen	0.143	30.531***	4.528
		Available potassium	0.302	4.667	0.302
		Available phosphate	1.459	6.49*	0.363
		Acid phosphatase	2.945	2.881	0.134
		Alkaline phosphatase	9.502*	13.519 *	224.487***
		Soil organic carbon	5.218	0.722	8.097*
		Available Lead	4.611	7830.509***	4.575
Buckwheat		Shoot length	4.99	52.37***	5.68
		Stem diameter	0.15	22.15**	2.43
		Fresh weight	7.98*	53.88***	9.60*
		Dry weight	20.65**	40.47***	19.18**
		Malondialdehyde	1.80	4.65	0.37
		Superoxide dismutase	3.06	7.81*	0.15
		Peroxidase	102.93***	0	40.39***
	Chlorophyll	Chlorophyll a	0.45	0.195	2.48
		Chlorophyll b	0.81	0.41	0.42
		Total Chlorophyll	0.86	0.01	1.82
	Lead	Whole buckwheat	1.15	334.55***	1.07

Values represent F-statistics. Significance levels are indicated as follows: **p*<0.05, ***p*<0.01, ****p* < 0.001. PLA: polylactic acid microplastics; Pb: lead; PLA × Pb, interaction between PLA and Pb treatments. Soil parameters include physicochemical properties, enzyme activities, and available lead concentration. Buckwheat parameters include growth metrics, oxidative stress indicators, photosynthetic pigment contents, and lead accumulation. Each treatment group comprised three biologically independent replicates (n=3).

Supplementary Table 3 Properties of buckwheat seedlings in the treatments

Treatments	Shoot length (cm)	Stem diameter (cm)	Fresh weight (whole plant) (g)	Dry weight (whole plant) (g)
CK	45.01±4.90a	3.21±0.10a	4.71±0.24a	0.60±0.09a
Pb	23.12±1.46c	2.91±0.09c	1.98±0.90c	0.23±0.10b
PLA	36.73±4.68b	3.12±0.08ab	3.61±0.08b	0.30±0.03b
Pb+PLA	21.54±3.95c	2.74±0.16bc	1.93±0.15c	0.17±0.02b
Treatments	MDA (nmol g $^{-1}$ FW^{-1})	POD (U g-1 FW $^{-1}$)	Pb (mg kg $^{-1}$)	SOD (U g^{-1} FW^{-1})
CK	66.24±3.05a	245.76±68.23bc	35.76±7.80c	159.11±32.99a
Pb	31.47±12.05c	108.66±11.88c	253.87±31.09b	264.04±27.19a
PLA	47.24±2.14b	345.01±87.45b	34.91±4.02c	229.15±28.01a
Pb+PLA	32.11±7.42c	834.18±98.09a	278.35±21.99a	221.89±38.14a
Treatments	Chlorophyll a (mg g^{-1})	Chlorophyll b (mg g^{-1})	Chlorophyll (mg g^{-1})	
CK	1.83±0.04a	1.15±0.11a	2.98±0.41a	
Pb	1.67±0.91a	1.12±0.60a	2.79±0.66a	
PLA	1.92±0.70a	0.78±0.40a	2.71±0.74a	
Pb+PLA	2.34±0.89a	1.08±0.12a	3.42±0.81a	

Data are mean±standard deviation (SD) (n=3). Different superscript letters in a column indicate statistically significant differences (Tukey's multiple range test *p*<0.05). SOD, Superoxide Dismutase; MDA, Malondialdehyde; POD, Peroxidase; FW, Fresh Weight; CK, control with no PLA-MPs and Pb; PLA, polylactic acid microplastics treatment with 2 g PLA-MPs kg^{-1} soil; Pb, Pb treatment with 2 g Pb kg^{-1} soil; Pb+PLA, Combined Pb and PLA-MPs treatment with 2 g Pb and 2 g PLA-MPs kg^{-1} soil.

Supplementary Table 4 Bacterial community and viral community differences *p*-values between treatments (PerMANOVA)

	Pairs	R^2	*p* value	*p* adjusted
Bacterial community	CK vs Pb	0.795	0.10	0.12
	CK vs Pb+PLA	0.851	0.10	0.12
	CK vs PLA	0.154	0.70	0.70
	Pb vs Pb+PLA	0.692	0.10	0.12
	Pb vs PLA	0.803	0.10	0.12
	Pb+PLA vs PLA	0.858	0.10	0.12
	All treatments	0.851	0.002	
	Pairs	R^2	*p* value	*p* adjusted
Viral community	CK vs Pb	0.491	0.10	0.12
	CK vs Pb+PLA	0.621	0.10	0.12
	CK vs PLA	0.373	0.10	0.12
	Pb vs Pb+PLA	0.312	0.20	0.20
	Pb vs PLA	0.55	0.10	0.12
	Pb+PLA vs PLA	0.543	0.10	0.12
	All treatments	0.600	0.001	

R^2 values indicate the proportion of variance explained by treatment group differences. Raw *p* values and adjusted *p* values are shown for all pairwise comparisons, alongside the overall PerMANOVA result across all treatments. PerMANOVA was performed based on Bray-Curtis dissimilarity matrices with 999 permutations. CK, control with no PLA-MPs and Pb; PLA, polylactic acid microplastics treatment with 2 g PLA-MPs kg^{-1} soil; Pb, Pb treatment with 2 g Pb kg^{-1} soil; Pb+PLA, Combined Pb and PLA-MPs treatment with 2 g Pb and 2 g PLA-MPs kg^{-1} soil.

Supplementary Table 5 Association of environmental factors with the beta-diversity of the bacterial and viral communities.

Bacterial community	Forward selection	Step	Variable	Adjusted R^2	△Adjusted R^2	F	*p* value
		1	APb	0.1373	+0.1373	2.7511	0.001***
		2	AK	0.1708	+0.0335	1.4042	0.047*
		3	ACP	0.1953	+0.0245	1.2735	0.083
	Envif		RDA1	RDA2	R^2	Pr(>r)	
		pH	-0.94791	-0.31853	0.8133	0.002**	
		AN	0.94217	0.33515	0.7304	0.007**	
		SOC	-0.57996	0.81465	0.147	0.49	
		TN	0.52896	0.84865	0.8233	0.001***	
		AK	0.40755	0.91318	0.9781	0.001***	
		APb	0.97931	0.20238	0.9903	0.001***	
		AP	-0.94612	-0.32383	0.3826	0.097	
		ACP	0.9871	-0.16013	0.2708	0.279	
		ALP	-0.86211	0.50673	0.1887	0.383	
Viral community	Forward selection	Step	Variable	Adjusted R^2	△Adjusted R^2	F	*p* value
		1	APb	0.3675	+0.3675	7.392	0.001***
		2	ALP	0.3845	+0.0170	1.2765	0.148
	Envif		RDA1	RDA2	R^2	Pr(>r)	
		pH	-0.99089	-0.13466	0.7411	0.005**	
		AN	0.9886	0.15057	0.6595	0.011*	
		SOC	-0.55648	0.83086	0.1953	0.373	
		TN	0.65484	0.75576	0.8128	0.001***	
		AK	0.6319	0.77505	0.9427	0.002**	
		APb	0.99996	0.00876	0.9562	0.004**	
		AP	-0.97905	-0.20361	0.3075	0.134	
		ACP	0.90498	-0.42546	0.3502	0.187	
		ALP	-0.59969	0.80023	0.1868	0.379	

*** $p<0.001$; ** $p<0.01$; * $p<0.05$. Environmental predictors of community composition were selected using forward selection based on adjusted R^2 and permutation tests (n = 999). Associations between ordination axes and environmental variables were further evaluated using the envfit function. For the bacterial community, only the first two variables (APb and AK) were retained in the final RDA model based on adjusted R^2 and $p<0.05$; for the viral community, only APb was retained in the final model based on adjusted R^2 and $p<0.05$.

Supplementary Table 6 Virome sequence assembly results

Sample	Total_base(Mb)	Total_num	Max_len	Min_len	N50	GC (%)	Read_used(%)
CK_1	37.44	16337	401119	300	12155	57.32	99.51
CK_2	36.76	16647	573797	300	11371	55.73	99.57
CK_3	43.19	15759	573798	300	14638	56.16	99.6
Pb_1	41.7	17238	574234	300	14664	58.52	98.94
Pb_2	39.84	17635	573794	300	10723	57.53	99.52
Pb_3	34.9	11730	401690	300	23431	58.33	99.67
PLA_1	65.52	38363	528111	300	4244	51.75	99.18
PLA_2	44.47	16089	491219	301	18226	58.69	99.5
PLA_3	44.65	16121	574136	300	18983	57.7	99.68
Pb+PLA_1	45.64	16593	574363	300	20716	58.92	99.54
Pb+PLA_2	34.27	16894	269079	300	10748	53.73	99.5
Pb+PLA_3	36.79	15217	401338	300	17093	58.68	99.58
MergedSample	20.99	7778	89229	300	5799	50.47	NA

Total_base, total number of sequence bases obtained by assembly; Total_num, number of contigs assembled; Max_len, maximum contig length; Max_len, maximum value of contig length; Min_len: minimum value of contig length; N50, N50 value; GC, average GC content of contigs; Read_used, utilization rate of reads during assembly. CK, control with no PLA-MPs and Pb; PLA, polylactic acid microplastics treatment with 2 g PLA-MPs kg^{-1} soil; Pb, Pb treatment with 2 g Pb kg^{-1} soil; Pb+PLA, Combined Pb and PLA-MPs treatment with 2 g Pb and 2 g PLA-MPs kg^{-1} soil.

Supplementary Table 7 Pairwise Fisher's exact tests with FDR correction on the number of lytic vs. temperate viruses among treatments

Comparison	Raw P value	Adjusted P (FDR)
CK vs PLA	0.530	0.796
CK vs Pb	0.109	0.654
CK vs Pb+PLA	0.768	0.921
PLA vs Pb	0.316	0.758
PLA vs Pb+PLA	1.000	1.000
Pb vs Pb+PLA	0.379	0.758

Raw *p* values and FDR-adjusted *p* values are shown for all pairwise comparisons. Statistical significance was assessed using Fisher's exact test, with multiple testing correction applied using the Benjamini–Hochberg false discovery rate (FDR) method. CK, control with no PLA-MPs and Pb; PLA, polylactic acid microplastics treatment with 2 g PLA-MPs kg^{-1} soil; Pb, Pb treatment with 2 g Pb kg^{-1} soil; Pb+PLA, Combined Pb and PLA-MPs treatment with 2 g Pb and 2 g PLA-MPs kg^{-1} soil.

Supplementary Table 8 Pairwise Fisher's exact tests with FDR correction on the number of viral auxiliary metabolic genes (AMGs) in functional categories.

Category	Comparison	Raw *p* value	Adjusted *p* (FDR)
Carbon Utilization	CK vs PLA	1.000	1.000
	CK vs Pb	0.228	0.343
	CK vs Pb+PLA	0.179	0.343
	PLA vs Pb	0.208	0.343
	PLA vs Pb+PLA	0.219	0.343
	Pb vs Pb+PLA	1.000	1.000
Organic Nitrogen	All comparisons	1.000	1.000
Miscellaneous	All comparisons	1.000	1.000

Raw *p* values and FDR-adjusted *p* values are shown for all pairwise comparisons within each AMG functional category, including carbon utilization, organic nitrogen metabolism, and miscellaneous functions. Statistical significance was assessed using Fisher's exact test, with multiple testing correction applied using the Benjamini–Hochberg false discovery rate (FDR) method. CK, control with no PLA-MPs and Pb; PLA, polylactic acid microplastics treatment with 2 g PLA-MPs kg^{-1} soil; Pb, Pb treatment with 2 g Pb kg^{-1} soil; Pb+PLA, Combined Pb and PLA-MPs treatment with 2 g Pb and 2 g PLA-MPs kg^{-1} soil.

Supplementary Table 9 Comparison of carboxylesterase (CarE) auxiliary metabolic genes (AMGs) with the PAZY Database

Contig	Auxiliary score	Amg flags
CK2_contig_10518-cat_1_4	1	MF
Pb3_contig_1082-cat_1_37	1	M
PLA3_contig_6091-cat_2_24	3	M
PLA2_contig_803-cat_1_7	1	MF
Pb+PLA2_contig_4540-cat_1_57	1	M
Pb+PLA1_contig_16258-cat_1_75	2	M
Pb+PLA3_contig_12270-cat_1_5	2	MF

Functional annotations of carboxylesterase and related enzymes across all contigs include: CE1 acetyl xylan esterase (EC 3.1.1.72); cinnamoyl esterase (EC 3.1.1.-); feruloyl esterase (EC 3.1.1.73); carboxylesterase (EC 3.1.1.1); S-formylglutathione hydrolase (EC 3.1.2.12); diacylglycerol O-acyltransferase (EC 2.3.1.20); trehalose 6-O-mycolyltransferase (EC 2.3.1.122). Auxiliary score) the score of auxiliary metabolic genes (ranging from 1 to 3). A score of 1 indicates that virus - characteristic genes are present on both sides, with the highest confidence level; a score of 2 indicates that a virus - characteristic gene is present on one side and a virus - like gene is on the other side, with a moderate confidence level; a score of 3 indicates that virus - like genes are present on both sides, with a low confidence level; Amg flags) tags for auxiliary metabolic genes. "M" indicates an auxiliary metabolic gene. "F" indicates that the auxiliary metabolic gene is located at the end of the sequence and has a length of less than 5 kb. CK, control with no PLA-MPs and Pb; PLA, polylactic acid microplastics treatment with 2 g PLA-MPs kg^{-1} soil; Pb, Pb treatment with 2 g Pb kg^{-1} soil; Pb+PLA, Combined Pb and PLA-MPs treatment with 2 g Pb and 2 g PLA-MPs kg^{-1} soil.

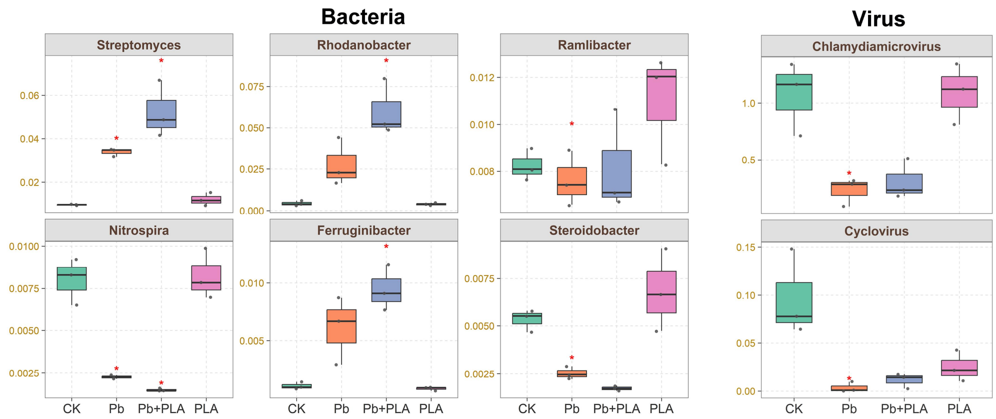

Supplementary Figure 1 Differential abundance analysis at the genus level with the R package ANCOM-BC (v. 2.0.2). Twenty genera with highest relative abundances across all samples were included in the analysis. *Statistically significant difference (p<0.05, n=3) compared with the control. CK, control with no PLA-MPs and Pb; PLA, polylactic acid microplastics treatment with 2 g PLA-MPs kg^{-1} soil; Pb, Pb treatment with 2 g Pb kg^{-1} soil; Pb+PLA, Combined Pb and PLA-MPs treatment with 2 g Pb and 2 g PLA-MPs kg^{-1} soil.

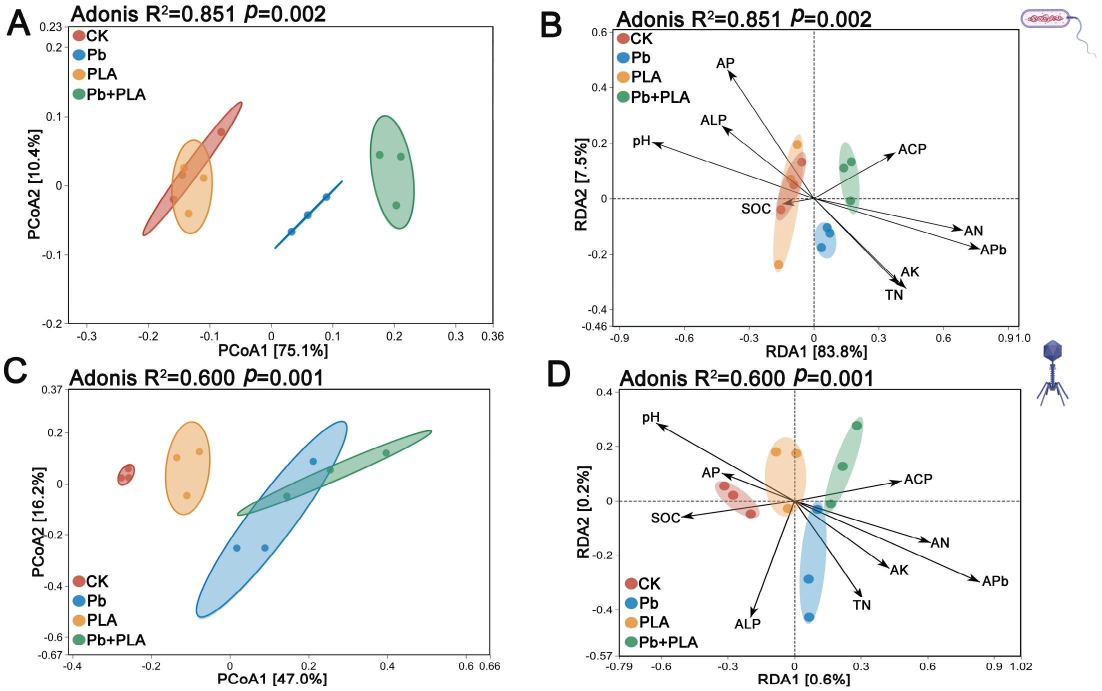

Supplementary Figure 2 Bacterial and viral communities in buckwheat rhizosphere soil. (A) Beta-diversity of the bacterial communities. **(B)** The association of environmental factors with the beta-diversity of the bacterial communities. **(C)** Beta-diversity of the viral communities. **(D)** The association of environmental factors with the beta-diversity of the viral communities. CK, control with no PLA-MPs and Pb; PLA, polylactic acid microplastics treatment with 2 g PLA-MPs kg^{-1} soil; Pb, Pb treatment with 2 g Pb kg^{-1} soil; Pb+PLA, Combined Pb and PLA-MPs treatment with 2 g Pb and 2 g PLA-MPs kg^{-1} soil.

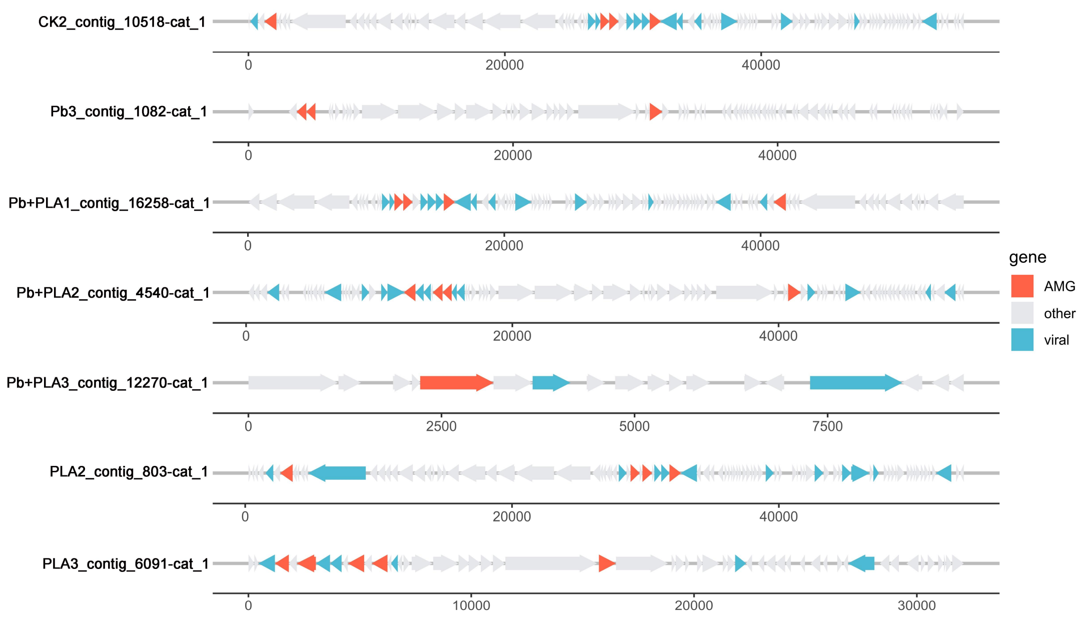

Supplementary Figure 3 Genome maps of viral contigs showing the location of carboxylesterase auxiliary metabolic genes (AMGs). Each track represents a single viral contig, with arrows indicating predicted open reading frames (ORFs) and their transcriptional orientation. Genes are colour-coded as follows: red, AMGs encoding carbohydrate esterases; blue, viral hallmark genes; grey, other genes with no assigned function. The x-axis indicates genomic position in base pairs. Contigs are derived from samples across all four treatment groups (CK, Pb, PLA, and Pb+PLA). CK, control with no PLA-MPs and Pb; PLA, polylactic acid microplastics treatment with 2 g PLA-MPs kg^{-1} soil; Pb, Pb treatment with 2 g Pb kg^{-1} soil; Pb+PLA, Combined Pb and PLA-MPs treatment with 2 g Pb and 2 g PLA-MPs kg^{-1} soil.

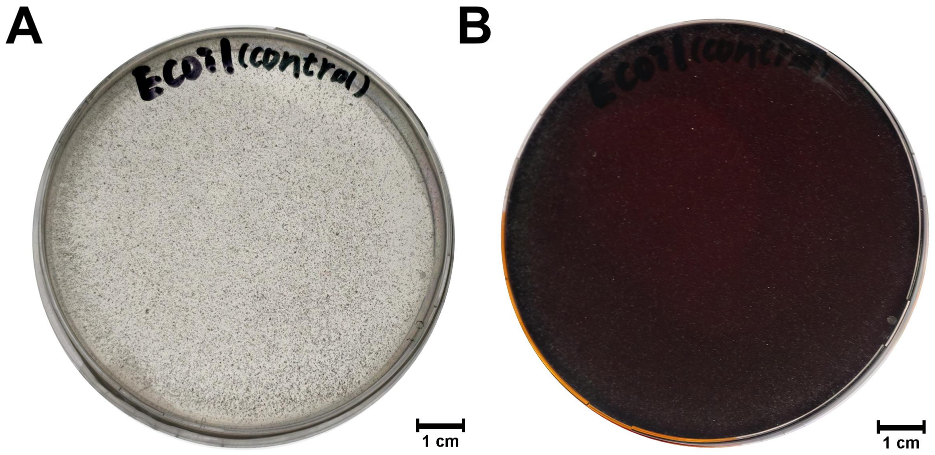

Supplementary Figure 4 Control plates illustrating the absence of halo formation in non-recombinant *E. coli*. Non-recombinant *E. coli* (host strain without the target gene) was streaked onto PLA-selective mineral salt medium (MSM) containing emulsified PLA (1.00 g L^{-1}) as the sole carbon source and incubated at 37 °C for 72 h. **(A)** Plates were photographed before and **(B)** after iodine–potassium iodide (I_2–KI) staining.

Supplementary References

1. Zhang, G. & Gong, Z. Soil Survey Laboratory Methods. Science Press, Beijing (2012).
2. Feng, X. et al. Microplastics change soil properties, heavy metal availability and bacterial community in a Pb-Zn contaminated soil. J. Hazard. Mater. 424, 127364 (2022).
3. Zhang, S. et al. Microplastic contamination in Chinese topsoil from 1980 to 2050. Sci. Total Environ. 955, 176918 (2024).
4. Dagari, M. S. & Badamasi, H. Lead (Pb) accumulation in plants grown on contaminated soil. Int. J. Sci. Res. Sci. Technol. 7, 401–405 (2020).
5. Wei, X. et al. Bacterial community in the buckwheat rhizosphere responds more sensitively to single microplastics in lead-contaminated soil compared to the arbuscular mycorrhizal fungi community. Ecotoxicol. Environ. Saf. 281, 116683 (2024).
6. Phillips, R. P. & Fahey, T. J. Tree species and mycorrhizal associations influence the magnitude of rhizosphere effects. Ecology 87, 1302–1313 (2006).
7. Chen, L. et al. Meta-analysis of impacts of microplastics on plant heavy metal(loid) accumulation. Environ. Pollut. 348, 123787 (2024).
8. Amjad, M. et al. Accumulation and translocation of lead in vegetables through intensive use of organic manure and mineral fertilizers with wastewater. Sci. Rep. 14, 12641 (2024).
9. Chen, S. et al. fastp: an ultra-fast all-in-one FASTQ preprocessor. Bioinformatics 34, i884–i890 (2018).
10. Bolger, A. M., Lohse, M. & Usadel, B. Trimmomatic: a flexible trimmer for Illumina sequence data. Bioinformatics 30, 2114–2120 (2014).
11. Li, D. et al. MEGAHIT: an ultra-fast single-node solution for large and complex metagenomics assembly via succinct de Bruijn graph. Bioinformatics 31, 1674–1676 (2015).
12. Gemayel, K., Lomsadze, A. & Borodovsky, M. MetaGeneMark-2: improved gene prediction in metagenomes. bioRxiv 500264 (2022).
13. Steinegger, M. & Söding, J. Clustering huge protein sequence sets in linear time. Nat. Commun. 9, 2542 (2018).
14. Hyatt, D. et al. Prodigal: prokaryotic gene recognition and translation initiation site identification. BMC Bioinformatics 11, 119 (2010).
15. Bushnell, B. BBMap: a fast, accurate, splice-aware aligner. Lawrence Berkeley National Laboratory, Berkeley, CA (2014).
16. Buchfink, B., Xie, C. & Huson, D. H. Fast and sensitive protein alignment using DIAMOND. Nat. Methods 12, 59–60 (2015).
17. Huson, D. H. et al. Integrative analysis of environmental sequences using MEGAN4. Genome Res. 21, 1552–1560 (2011).
18. Szpara, M. L., Tafuri, Y. R. & Enquist, L. W. Preparation of viral DNA from nucleocapsids. J. Vis. Exp. 54, 3151 (2011).
19. Li, H. & Durbin, R. Fast and accurate short read alignment with Burrows-Wheeler transform. Bioinformatics 25, 1754–1760 (2009).
20. Nayfach, S. et al. CheckV assesses the quality and completeness of metagenome-assembled viral genomes. Nat. Biotechnol. 39, 578–585 (2021).
21. Guo, J. et al. VirSorter2: a multi-classifier, expert-guided approach to detect diverse DNA and RNA viruses. Microbiome 9, 37 (2021).

22. Li, W. & Godzik, A. Cd-hit: a fast program for clustering and comparing large sets of protein or nucleotide sequences. Bioinformatics 22, 1658–1659 (2006).
23. Jiang, J. et al. Virus classification for viral genomic fragments using PhaGCN2. Brief. Bioinform. 24, bbac505 (2023).
24. Simmonds, P. et al. Changes to virus taxonomy, the international code of virus classification and nomenclature, and the ICTV statutes ratified by the International Committee on Taxonomy of Viruses (2025). Arch. Virol. 171, 23 (2025).
25. Liu, X. Isolation and degradation characteristics of polyethylene and polyvinyl alcohol degrading bacteria in soil. Master's thesis, Shandong Agricultural University, Taian, China (2023).
26. Nakamura, K. et al. Purification and characterization of an extracellular poly(L-lactic acid) depolymerase from a soil isolate, Amycolatopsis sp. strain K104-1. Appl. Environ. Microbiol. 67, 345–353 (2001).
27. Teeraphatpornchai, T., Nakajima-Kambe, T. & Shigeno-Akutsu, Y. Isolation and characterization of a bacterium that degrades various polyester-based biodegradable plastics. Biotechnol. Lett. 25, 23–28 (2003).
