# Supplementary material for: Microplastic and lead shift microbiomes enriching viral auxiliary metabolic genes for potential polylactic acid degradation
Source: Commun Biol. 2026 May 7;9:949. doi: 10.1038/s42003-026-10162-7 (PMC13365211; doi:10.1038/s42003-026-10162-7)
Supplement: Supplementary file 3 — Description of Additional Supplementary files [file 42003_2026_10162_MOESM3_ESM.pdf]

Description of Additional Supplementary Files

Supplementary Data 1-This table contains the data of figure1-5 and supplementary table3

Supplementary Data 2-

i) Metagenomic sequencing data statistics

ii) Distribution of host-virus links

iii) Effects of PLA, Pb, and their interactions on gene abundances in KEGG pathways and Pb resistance (Bacmet) based on a Two-way ANOVA analysis
