# Supplementary material for: Microplastic and lead shift microbiomes enriching viral auxiliary metabolic genes for potential polylactic acid degradation
Source: Commun Biol. 2026 May 7;9:949. doi: 10.1038/s42003-026-10162-7 (PMC13365211; doi:10.1038/s42003-026-10162-7)
Supplement: Supplementary file 6 — reporting summary [file 42003_2026_10162_MOESM6_ESM.pdf]

nature portfolio

Corresponding author(s): yunfu Gu COMMSBIO-25-11941

Last updated by author(s): 7 April 2026

Reporting Summary

Nature Portfolio wishes to improve the reproducibility of the work that we publish. This form provides structure for consistency and transparency in reporting. For further information on Nature Portfolio policies, see our Editorial Policies and the Editorial Policy Checklist.

Statistics

For all statistical analyses, confirm that the following items are present in the figure legend, table legend, main text, or Methods section.

n/a	Confirmed	
☐	☒	The exact sample size (*n*) for each experimental group/condition, given as a discrete number and unit of measurement
☐	☒	A statement on whether measurements were taken from distinct samples or whether the same sample was measured repeatedly
☐	☒	The statistical test(s) used AND whether they are one- or two-sided<br>*Only common tests should be described solely by name; describe more complex techniques in the Methods section.*
☐	☒	A description of all covariates tested
☐	☒	A description of any assumptions or corrections, such as tests of normality and adjustment for multiple comparisons
☐	☒	A full description of the statistical parameters including central tendency (e.g. means) or other basic estimates (e.g. regression coefficient) AND variation (e.g. standard deviation) or associated estimates of uncertainty (e.g. confidence intervals)
☐	☒	For null hypothesis testing, the test statistic (e.g. F, t, r) with confidence intervals, effect sizes, degrees of freedom and P value noted<br>*Give P values as exact values whenever suitable.*
☒	☐	For Bayesian analysis, information on the choice of priors and Markov chain Monte Carlo settings
☐	☒	For hierarchical and complex designs, identification of the appropriate level for tests and full reporting of outcomes
☐	☒	Estimates of effect sizes (e.g. Cohen's *d*, Pearson's *r*), indicating how they were calculated

Our web collection on statistics for biologists contains articles on many of the points above.

Software and code

Policy information about availability of computer code

Data collection	Data were collected from independent biological replicates for each treatment group. Each replicate represented a separate experimental unit. No repeated measurements were performed on the same sample, and no data points were excluded from the study.
Data analysis	Data processing, statistical analyses, and visualization were performed using publicly available software, including Fastp, Trimmomatic, MEGAHIT, MetaGeneMark, Prodigal, CD-HIT, DIAMOND, MEGAN, CheckV, VirSorter2, PhaGCN2, and R (v3.6.2) with relevant packages. Additional analyses were conducted using IBM SPSS Statistics (v26), GraphPad Prism (v8.0), and Origin (v2023). No custom code was developed for this study.

For manuscripts utilizing custom algorithms or software that are central to the research but not yet described in published literature, software must be made available to editors and reviewers. We strongly encourage code deposition in a community repository (e.g. GitHub). See the Nature Portfolio guidelines for submitting code & software for further information.

Data

Policy information about availability of data

All manuscripts must include a data availability statement. This statement should provide the following information, where applicable:

- Accession codes, unique identifiers, or web links for publicly available datasets
- A description of any restrictions on data availability
- For clinical datasets or third party data, please ensure that the statement adheres to our policy

Provide your data availability statement here.

The raw bacterial and viral metagenomic sequencing data generated in this study have been deposited in the NCBI Sequence Read Archive (SRA) under BioProject accession numbers PRJNA1266835 and PRJNA1266889. All datasets are publicly accessible and can be accessed through the NCBI BioProject database.

Research involving human participants, their data, or biological material

Policy information about studies with human participants or human data. See also policy information about sex, gender (identity/presentation), and sexual orientation and race, ethnicity and racism.

Reporting on sex and gender	This study did not involve human participants, human data, or human biological material.
Reporting on race, ethnicity, or other socially relevant groupings	Not applicable. This study did not involve human participants, and no variables related to race, ethnicity, or other socially constructed groupings were collected or analyzed.
Population characteristics	Not applicable. No human population was involved in this study.
Recruitment	Not applicable. No human participants were recruited for this study.
Ethics oversight	Not applicable. Ethical approval and informed consent were not required as this study did not involve human participants or human-derived materials.

Note that full information on the approval of the study protocol must also be provided in the manuscript.

Field-specific reporting

Please select the one below that is the best fit for your research. If you are not sure, read the appropriate sections before making your selection.

☐ Life sciences ☐ Behavioural & social sciences ☒ Ecological, evolutionary & environmental sciences

For a reference copy of the document with all sections, see nature.com/documents/nr-reporting-summary-flat.pdf

Ecological, evolutionary & environmental sciences study design

All studies must disclose on these points even when the disclosure is negative.

Study description	This study used a controlled pot experiment with a fully factorial design to investigate the individual and combined effects of lead (Pb) and polylactic acid microplastics (PLA-MPs) on buckwheat rhizosphere bacterial and viral communities. Four treatments were applied: control (CK), Pb, PLA-MPs, and Pb+PLA, each with three independent biological replicates. Soil microbial community composition, functional gene profiles, and plant physiological traits were quantified.
Research sample	The research samples consisted of agricultural soil collected from Huili County, Sichuan Province, China, and buckwheat (Fagopyrum esculentum) grown under greenhouse pot conditions. Rhizosphere soil, bulk soil, and plant tissues were collected for microbial, physicochemical, and physiological analyses.
Sampling strategy	Three independent pots per treatment were used as biological replicates. Sample sizes were determined based on previous soil microbiome and viromics studies using similar experimental designs, rather than formal power calculations.
Data collection	Soil and plant samples were collected at harvest following a standardized protocol. Rhizosphere soil was collected by gently shaking roots and manually retrieving soil adhering to the root surface. Bulk soil and plant tissues were collected simultaneously. All measurements were conducted following established protocols as described in the Methods and Supplementary Methods.
Timing and spatial scale	The pot experiment was conducted under greenhouse conditions in Sichuan Agricultural University. After 60 days, Soil sampling was performed at the end of the growth period. All samples were collected from the same experimental location and time point.
Data exclusions	No data were excluded from the analyses
Reproducibility	All experiments were performed with independent biological replicates. Standardized experimental and analytical protocols were applied consistently across treatments (see method and supplementary information)

Randomization	Samples were randomly assigned to treatment groups.
Blinding	Blinding was not applied as the experimental design did not require subjective assessment.

Did the study involve field work? ☐ Yes ☒ No

Reporting for specific materials, systems and methods

We require information from authors about some types of materials, experimental systems and methods used in many studies. Here, indicate whether each material, system or method listed is relevant to your study. If you are not sure if a list item applies to your research, read the appropriate section before selecting a response.

Materials & experimental systems

n/a	Involved in the study
☒	☐ Antibodies
☒	☐ Eukaryotic cell lines
☒	☐ Palaeontology and archaeology
☒	☐ Animals and other organisms
☒	☐ Clinical data
☒	☐ Dual use research of concern
☐	☒ Plants

Methods

n/a	Involved in the study
☒	☐ ChIP-seq
☒	☐ Flow cytometry
☒	☐ MRI-based neuroimaging

Plants

Seed stocks	Seeds of buckwheat (Fagopyrum tataricum L.) were obtained from a certified local agricultural supplier in Sichuan Province, China. Seeds used in this study were commercially available, non-transgenic, and commonly cultivated varieties.
Novel plant genotypes	This study did not involve the generation or use of novel plant genotypes, genetically modified plants, or mutant lines.
Authentication	Plant species identity was based on standard morphological characteristics and seed source information provided by the supplier. No additional molecular authentication was required for this study.
