# Supplementary material for: Microplastic and lead shift microbiomes enriching viral auxiliary metabolic genes for potential polylactic acid degradation
Source: Commun Biol. 2026 May 7;9:949. doi: 10.1038/s42003-026-10162-7 (PMC13365211; doi:10.1038/s42003-026-10162-7)
Supplement: Supplementary file 1 — Transparent Peer Review file [file 42003_2026_10162_MOESM1_ESM.pdf]

nature portfolio

Peer Review File

Microplastic and Lead Shift Microbiomes, Enrich Viral Auxiliary Metabolic Genes for Potential Polylactic Acid Degradation

Corresponding Author: Professor Yunfu Gu

This file contains all reviewer reports in order by version, followed by all author rebuttals in order by version.

Attachments originally included by the reviewers as part of their assessment can be found at the end of this file.

Version 0:

Reviewer comments:

Reviewer #1

(Remarks to the Author)
The present study is well-written and eloquently describe an underexplored subject in terrestrial microplastic and heavy metal pollution. The impact of viruses on the metabolization and adaption to emerging pollutants builds a novel perspective and add substantial value to the existing knowledge. The addition of experimental data on biodegradation is a great indication for the quality of the work and I personally appreciate these data in times were sequencing data are very dominant in the plastisphere research, even though the applied method has certain limitations (s. below).

Despite my recommendation for publishing this great work I would ask the authors to generally give more explanations on the extent and impact of the two applied pollutants Pb and PLA and their projected impact in the future and why a co-contamination is likely in which types of environments?
At certain paragraphs especially in the discussion the authors should add more cross references for their own data.

My personal expertise does not qualify me to comment too much on certain statistical analyses and the elaborated description of viral sequence evaluation.

In the following there are certain comments and suggestions that I kindly ask to work through before publication.

l. 16 Could use „authors“ as there are two persons mentioned.
ll.21,22 The statement appears to be very strong and I would disagree that Biodegradable microplastic and heavy metals are that often-co-occurring.
l. 25 Would leave out “in a controlled trial”

llf. 42 Would suggest to add that PLA degrades extremely slow under environmental conditions and thus PLA microplastic pollution can be an emerging threat, even though it is biodegradable.
l. 45 Could you elaborate more how this “passive sampling” influence heavy metal mobility.
llf. 79 I would rephrase the last hypothesis as it is counter-intuitive to label PLA degradation as disruption and would focus more on how the interplay of both contaminants acts in terrestrial ecosytems.
Remark to the introduction:
I am missing information about what streams of contamination do you have in mind. Describe in which amounts PLA and Pb are entering soil systems to underline the importance of both pollutants. If possible, it would be helpful to give estimates of the quantity of pollutants that is expected to enter the environment. Either on a global scale or in the distinct region you have set the study.
ll 86, 87 please make sure if kg soil for fresh/ wet soil or dry soil
l 89 Is the agricultural soil in active agriculture or just arable land? As you describe the elimination of litter and stones it sounds like inactive agricultural soil.

l. 125 Would appreciate to name the author of the method
l. 139 de novo in italics
llf. 154-159 Could you elaborate more on how you combined the two software results and if the “High-confidence sequences” were from Virsorter or from CheckV.
llf. 172 Would appreciate if you add a reference here.
llf. 196 Please elaborate more on how you combined the different tools together for annotation
llf. Chapter 2.9 I would expect to have read the CarE identification in the results part and not in the M&M part. Would suggest to rephrase and focus less on this specific gene.
Llf. 229 I like the concept, but would suggest to make an additional chapter with the experimental validation of CarE as it would help the readers to follow.
For some companies you give the city name for others you don´t. Please harmonize
l. 235 Please give more information about the origin of PLA as well as particle size etc. and what exactly do you mean by emulsified PLA (Supplement)
l. 236 I like the staining idea! Is there a reference for it or have you come up with the idea by yourself? Could you briefly describe what exactly it is staining? Like PLA Monomers or something else?
l.247 Where parametric assumptions met for all displayed data or was it necessary to do other tests for certain data?
Llf. 259 Consider to make a separate chapter for RDA plot instead of having such an elaborated statistical analysis chapter
l. 269 I personally prefer to have one or two descriptive sentences before starting with the results part right away. Also, I would suggest highlighting that BOTH treatments with Pb have effects to make it more easy to grasp your information.
Llf. 277 Would suggest to reorder the results and focus for instance first only on macroscopic plant growth than contents and lastly the activities.
Further please give a brief explanation what do you want to examine with MDA and POD activity or what you can retrieve from these data.
ll. 288,289 What exactly are you describing with the sign “~”
Your bacterial nomenclature is outdated as for instance Proteobacteria are named Pseudomonadota now. Please adjust all names in the text and in the figures accordingly.
https://www.microbiologyresearch.org/content/journal/ijsem/10.1099/ijsem.0.005056
https://www.nature.com/articles/s41522-024-00494-9
check the work from Oren et al.
l. 307 I am not a virologist, just double check if there have been changes in the nomenclature for viruses in recent years
l. 311 there is a space missing between Fig. and 1D please recheck these minor typos in the entire manuscript.
l. 316 I suggest to change the wording and do not use correlate as this is not well fitting in this context.
l. 326 Could you explain why exactly you chose the phylum-family link pattern and not phylum order etc.?
ll. 330,331 I have never done these links by myself but I wonder if there is kind of a variation between your replicates as you do not give any value variation for different treatments.
Would suggest to give a brief elaboration of what you want to show with these data.
l. 337 Not familiar with these types of analysis, but why using first a Scheirer-Ray-Hare test and here a Fisher test?
l. 338 Why is this finding notably? Please elaborate why you highlighted these. Are you referring with Chordata to the macroscopic animal group because I have not found a bacterium phylum named Chordata? Please clarify
l. 341 Why should a virus interact with a different host when the treatment changes?
l. 346 if you highlight level2 category please make sure to explain what is the difference to other levels and why you use level2
l. 347 Is the list done only with Bacmet or also manually curated or could you name a reference for this? I just wonder as I am not aware how the function “Drug resistance: Antineoplastic” and others are related to Pb tolerance.
L. 350 I am concerned about the label “relative abundance” as in Table S10 a treatment would have more than 100% relative abundance. Please clarify what the values in Table S10 are meaning and what they mean for your results.
It is up to you, but I would suggest to highlight some Pb/ PLA related damage potentials to your description. For instance, “Replication and repair” is dramatically increased in Pb treatments as well as Aging. I think you mention them later in the discussion, too.
l. 363 I think you could mention that overall, the effects of the treatments on viral functions appear to be much lower compared to the changes induced in bacterial function, right?
l.382 Any hints of enhanced quantities in comparison to non-PLA treatments?
l-389 If talking about multi-database name them or at least add references.
Llf. 386 Maybe you could rename the long gene names for simplification and define them in the supplement whenever useful.
l. 397 Is it really the CarE enzyme activity. As I understood it, your enzyme assay is quite unspecific, thus I would argue to rephrase it.
l.414 S. above please explain more what POD activity indicates
l. 417 I think it is even more fascinating that, the Pb content in PLA+Pb in soil is around 4,5% higher than in the Pb only incubation, though in the plant the Pb content is 9% higher in PLA+Pb than in the Pb only incubation. This further underline your hypothesis, that PLA MP enhances Pb mitigation.
Overall, you are missing some data references in this part of the discussion, please add cross references to your data
l. 452 Is there anything known about virus scavenging on MP surfaces and thus shadowing effects?
l. 457 Maybe a bit off topic, but do you have any information about the vitality of the bacterial community when coping with such high Pb concentrations? Something like live-dead staining etc.? Might be useful to check for these and correlate with lytic and temperate viral lifestyles.
Llf. 474 I disagree with your statement of PLA buffering Pb disruptive effects, as the total number of links was as low in PLA+Pb as in Pb only and just the distinct link number is changed. Further, I would ask to tone down the statement of “PLA contributing to ecosystem resilience” as this might lead to wrong conclusions when focusing on the entire ecosystem.

l.485 Please rephrase as hard to grasp what you want to say. And the Cadmium came a bit out of nowhere, please write the full name as this can easily be misunderstood as typo.
l.491 add crossference to your data
llf. 500 Would suggest to screen the literature if there are reports on N-cycling and Pb contamination.
l. 508 Would rephrase “viral N metabolism” as this would indicate a more active role of viruses.
Llf.528 I would tone down the paragraph as you do not have direct links to PLA degradation and you might overinterprete your esterase activity assay. I suggest to either convincingly argue that the assays are indeed indicating PLA degradation or be less enthusiastic about your conclusion. As the halos might be induced by defragmentation and not by mineralization. You have not provided information about actual monomers/ CO2 derived from PLA and not mentioned the content of small oligomeric PLA fractions or changed molecular masses. According to Sander et al. Biodegradation should be solely defined as the conversion of Bioplastic to CO2 or CH4.
Overall, I would like to have some hypothesize-like explanations why viruses should have an “interest” in enhancing PLA degradation in rhizosphere soil.
l. 591 rephrase as you have not done any toxicity assays in the study and the grammar is poor.
Figure 1
I would ask to not make the 20 most abundant genera having a total relative abundance of 1, but to adjust them in a way that the reader can see what is their proportion on the total relative abundance. So either add “others” or adjust the y-axis for improved visibility.
The labeling is very small, please use larger font size.
I am wondering why you have not indicated the phylum Deinococcus-Thermus in figure 2 and not having it in figure 1. Is there a missing “others” bar or something similar?
Figure 2
Very small font size, please rearrange and increase visibility.
Figure 3
Very small font size at some sections. Consider making two figures and increase font size substantially
In 3C Would suggest to order your pie plot from highest percentage to lowest, as this unordered visualization makes it counterintuitive to grasp the information.
3A Would suggest to put the Standardized gene abundance bar in the lower-right hand corner of the Heatmap. Further highlight the KEGG Level 1 categorization more (make the bars bigger for instance)
3C Include x-axis legend
Figure 4
Increase the font size and consider putting B and C below the plasmid map to increase visibility.
Further, I kindly ask to add also a picture of a plate with a non-recombinant E. coli with and without staining to clarify the halo formation. With bigger size for the images would be appreciated.

Supplement
Note: Is the Pb content of the tested soil in a normal range or rather high? If it has something like a Pb priming the results might be influenced.
Please clarify what exactly do you consider rhizosphere soil and how you obtained it. If available add a picture. Have there been separation methods to fractionate roots from soil or have you mixed root material together with surrounding soil etc.

In the paragraph "determining soil properties" is a space missing before “[3]”
The plant lead content examination should be described more elaborately and also why an acid digestion is necessary and add a reference.

In general please be more elaborate on the whole degradation assay and what you can retrieve from the results and what limitations do you face

Figure S2
Sometimes the end values of the axis were mentioned like 0.72 and sometimes not. Please harmonize. Further, the font sizes seem to be different. Please harmonize. Increase font size in general.
Please double check if the axis in S2D are correctly labelled as I think 0.2% and 0.6% appear to be quite low and if this is correct please mention the low coverage of the analysis in the text.
Table S10 /S11
I personally do not like the table as mentioned before. It is not clear what the values are meaning and I would suggest to add CK values to simplify the understanding of the data.

Overall great work!
Stephan Rohrbach

Reviewer #2

(Remarks to the Author)
Attached accept with minor revisions

Reviewer #3

(Remarks to the Author)

1. Line 86: The authors should clarify the rationale for selecting the applied concentrations of PLA microplastics and Pb. In particular, a concentration of 2 g kg^{-1} appears relatively high. Please discuss whether these levels are environmentally relevant and to what extent the observed effects may reflect high-dose responses rather than conditions representative of real agricultural soils. The implications for field relevance should be addressed.
2. Line 88: The rationale for selecting buckwheat as the model plant should be explained more clearly. Specifically, the authors should justify its relevance in terms of human diet, agricultural importance, and potential implications for public health or food security.
3. Line 92: The authors should briefly explain what each measured soil, plant, and biochemical indicator represents and its ecological or biological implications, rather than simply listing the parameters. This clarification should be applied consistently throughout the Methods section to improve methodological transparency.
4. Methods section (general): The Methods section is overly detailed. The authors are encouraged to retain only essential methodological information in the main text and move extensive procedural details to the Supplementary Materials, in line with the journal's formatting and readability expectations.
5. Results section: P-values should be consistently formatted as lowercase italic p throughout the Results section.
Results vs. Discussion:
6. The authors should more clearly separate the Results and Discussion sections. The Results should be limited to objective descriptions of the data without mechanistic interpretation, while the Discussion should avoid repeating experimental results. In the current version, there is substantial overlap between these sections, with repeated descriptions of results appearing in the Discussion.
7. Depth of Discussion (e.g. Section 4.1): The discussion remains relatively superficial. For example, in Section 4.1, although comparisons with previous studies are provided, there is limited in-depth analysis of why the observed results occurred and what factors may explain similarities or discrepancies with earlier findings. A deeper mechanistic and contextual interpretation is needed.

Version 1:

Reviewer comments:

Reviewer #1

(Remarks to the Author)
Thank you very much for working through my suggestions and and your positive resonance. I appreciate your improvements and wish you all the best

Open Access This Peer Review File is licensed under a Creative Commons Attribution 4.0 International License, which permits use, sharing, adaptation, distribution and reproduction in any medium or format, as long as you give appropriate credit to the original author(s) and the source, provide a link to the Creative Commons license, and indicate if changes were made.
In cases where reviewers are anonymous, credit should be given to 'Anonymous Referee' and the source.
The images or other third party material in this Peer Review File are included in the article's Creative Commons license, unless indicated otherwise in a credit line to the material. If material is not included in the article's Creative Commons license and your intended use is not permitted by statutory regulation or exceeds the permitted use, you will need to obtain

permission directly from the copyright holder.
To view a copy of this license, visit https://creativecommons.org/licenses/by/4.0/

Point-by-point Response to Reviewers

We would like to express our sincere gratitude to the editor and reviewers for their insightful and constructive comments. These suggestions have been instrumental in enhancing the quality of our manuscript. We have carefully revised the manuscript to address all raised concerns, and our point-by-point responses are detailed below. All changes in the manuscript are documented using the *Track Changes* feature, and we have also provided a 'clean' version with modified text *highlighted (grey)*. Please note that due to the Track Changes mode, line numbering in the manuscript may appear discontinuous; therefore, please refer to the line numbers indicated in my responses for accuracy. Once again, we deeply appreciate your time and valuable feedback.

From Editor:

1. Justification the environmental relevance and co-occurrence of applied Pb and PLA concentrations, especially the high PLA dose is required.
**Response:** Thank you for this important suggestion. We have revised the Introduction and Methods sections to clarify the rationale for the selected doses and their ecological context. The applied Pb concentration (2 g kg^{-1} soil) was chosen to represent a strong contamination scenario commonly used in pot and microcosm studies to elicit measurable biological responses and mechanistic insights, while remaining within the range reported for highly contaminated agricultural or industrially impacted soils. Importantly, the background soil used in this study contained Pb levels well below the national risk screening value (GB 15618–2018), minimizing potential Pb priming effects. And for the PLA microplastic concentration (2 g kg^{-1} soil), was intentionally selected to simulate a plausible future accumulation scenario under intensive and repeated application of biodegradable plastic films in agriculture. Recent large-scale assessments indicate rapidly increasing microplastic loads in croplands, and biodegradable polymers such as PLA are expected to become increasingly prevalent. Thus, the selected PLA dose allows us to explore potential ecological interactions between bio-MPs and lead under a high-exposure. Corresponding clarifications have been added to the revised **<u>Supplementary Material (Line 47-</u>**

**49).**
2-A clear explanation of the rationale for choosing buckwheat and the ecological/biological meaning of all measured indicators is needed.
**Response:** Thanks for the suggestion. We have clarified the rationale for selecting buckwheat in the revised Introduction **(Line 134-138)**, and add the meaning of all measured indicators in Method section **(Line 177-196).** Specifically, we now note that buckwheat is well documented to tolerate and accumulate heavy metals and has therefore been proposed as a model crop for studying plant responses to metal contamination. At the same time, its response to emerging pollutants such as microplastics remains poorly understood, making it a relevant and informative system for investigating the combined effects of Pb and PLA microplastics.
3. Improve separation between Results and Discussion, avoiding repetition and strengthening mechanistic interpretation.
**Response:** Thank you for the suggestion. In the revised manuscript, we have carefully improved the separation between the **Results and Discussion sections**. Redundant explanations previously repeated in both sections were removed.
4. Avoid overinterpretation, particularly regarding PLA degradation, buffering effects, and ecosystem resilience.
**Response:** Thank you for this important comment. We carefully re-evaluated the manuscript to avoid overinterpretation, particularly regarding PLA degradation, buffering effects, and ecosystem-level implications. In the revised version, statements referring to PLA degradation were consistently rephrased to emphasize hydrolytic activity toward ester-containing substrates and potential involvement in initial depolymerization steps. Claims regarding buffering effects were toned down and restricted to specific microbial and viral responses supported by the data.
5. Streamline the Methods section and move excessive procedural details to supplementary Materials.
**Response:** Thank you for this helpful suggestion. We have thoroughly revised the Methods section. Specifically, we have streamlined the main text by retaining only the essential methodological framework and core workflows, while moving extensive procedural details to the Supplementary Materials.
6. Clarify statistical approaches, assumptions, data normalization, and interpretation of relative abundance values.
**Response**: Thank you for the suggestion. We have revised the Method and Statistical Analyses section to explicitly clarify the normalization strategies and interpretation of relative abundance data for bacterial taxa, viral taxa, and functional genes.
7. Update and harmonize nomenclature, references, formatting, and terminology throughout the manuscript.
**Response:** Thank you for the suggestion. We have carefully revised the manuscript to update and harmonize nomenclature, references, formatting, and terminology throughout the text.

Specifically, taxonomic names were standardized according to the latest ICNP (for bacteria) and ICTV guidelines (for viruses), references were checked and updated where necessary, and formatting inconsistencies were corrected to ensure consistency across the manuscript and Supplementary Materials.

8. Improve figures and supplementary materials for readability, consistency, and clearer data representation.

Response: Thank you for pointing this. All figures in the main text and Supplementary Materials (Fig. S2) were revised to improve readability and clarity. Specifically, all the font sizes were increased, panel layouts were adjusted, and figure elements (legends, axis labels, and annotations) were harmonized for consistency. Where appropriate, complex figures were reorganized or separated to facilitate clearer data presentation. These revisions were made in accordance with the reviewer's suggestions.

Revised figure and what changes were made can be seen at below:

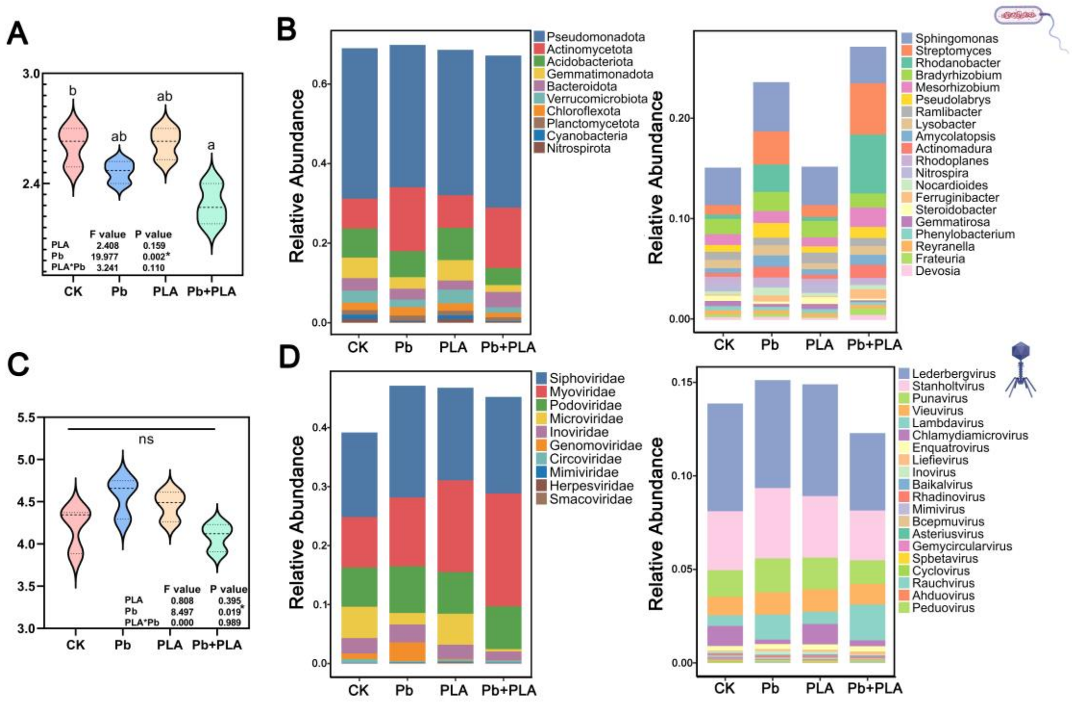

Revised_Figure 1, Change: We have revised the presentation of microbial relative abundance. Previously, the total abundance was normalized to 1 (100%) within each sample; however, we now present the non-normalized abundances. This change better illustrates the specific fluctuations of different genera across various treatments, as the reviewer's suggestion, and we used larger font size.

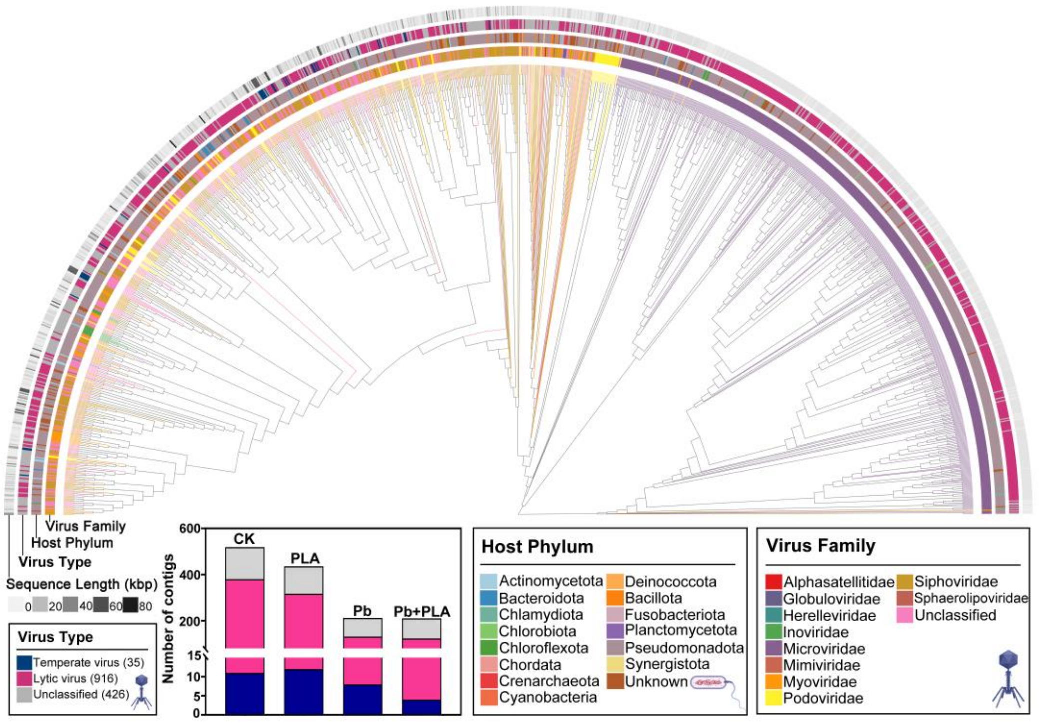

Revised_Figure 2, Change: The display information at the bottom of the original figure, including viral lifestyle, virus family, and host phylum, has been reformatted and the font size has been increased for better clarity.

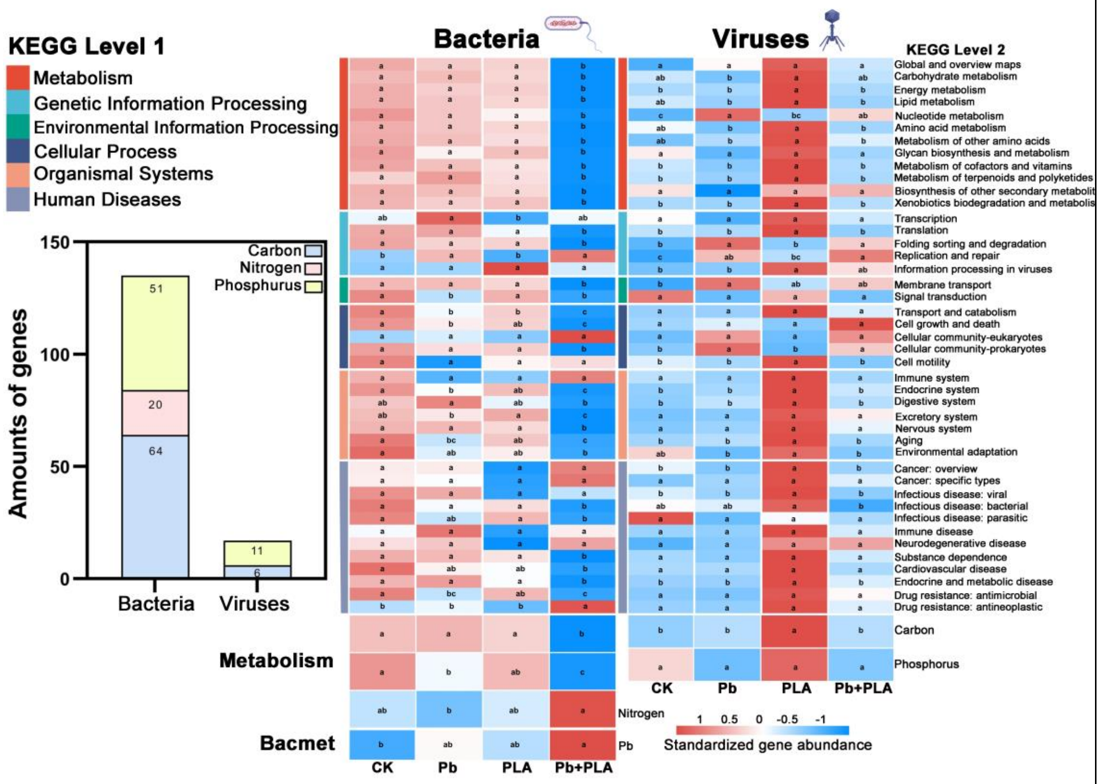

Revised_Figure 3, Change: The original Fig 3 has been split into two separate display items (Fig 3 and Fig 4). The updated Fig 3 now presents the functional gene variations in bacterial and viral communities, while the new Fig 4 focuses on the characteristics of viral AMGs.

Furthermore, the bar chart in the bottom-left corner of Fig 3 has been enlarged for better visibility, and the font size throughout both figures has been increased to improve readability.

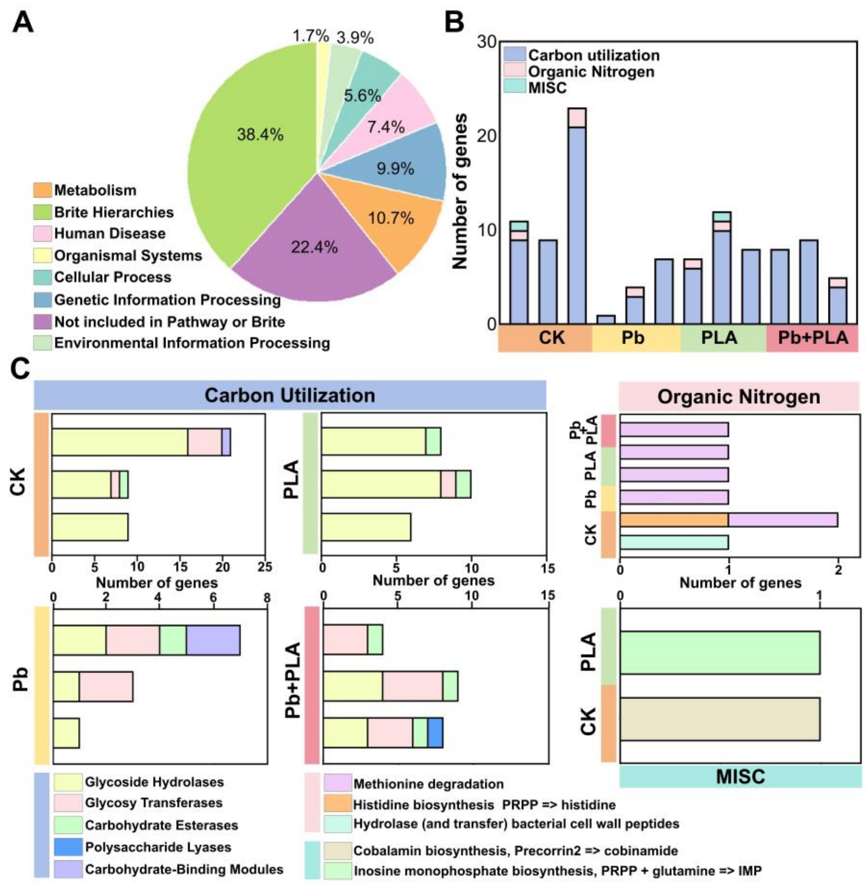

Revised_Figure 4, Change: Following the reviewer's suggestion to 'order your pie plot from highest percentage to lowest', we have updated the pie chart in Panel A. Additionally, we reformatted Panel C by enlarging all font sizes (including both X and Y axes) and supplementing the information on the X-axis.

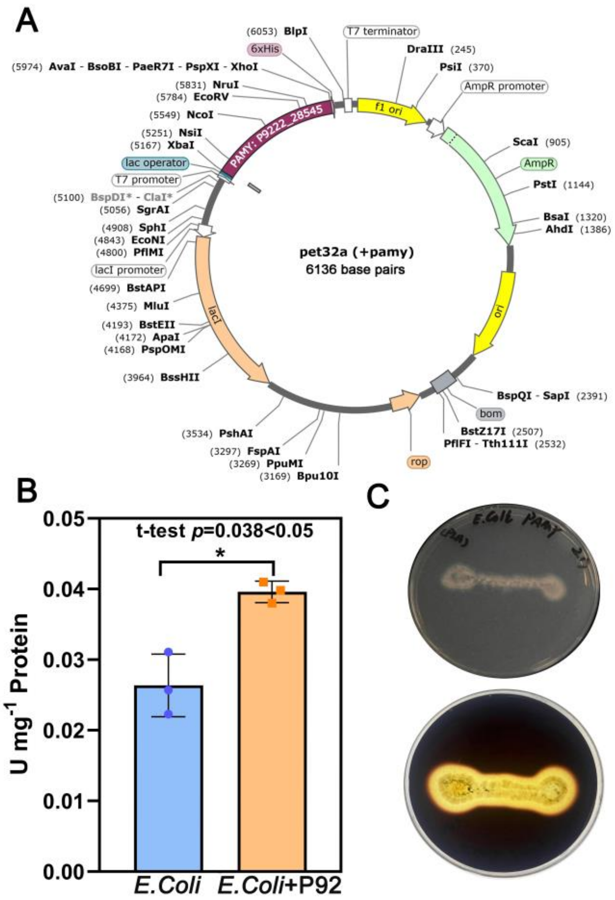

Revised_Figure 5, Change: The layout was updated by moving Panels B and C below Panel A. For the enzyme activity bar graphs in Panel B, individual replicate points were added to satisfy the requirement for showing data distribution. A new control image, Supplementary Fig S4 (see below), has been added to provide large-scale photographs of non-recombinant *E. coli* plates (with and without staining) to clarify halo formation, as requested by the reviewer#1

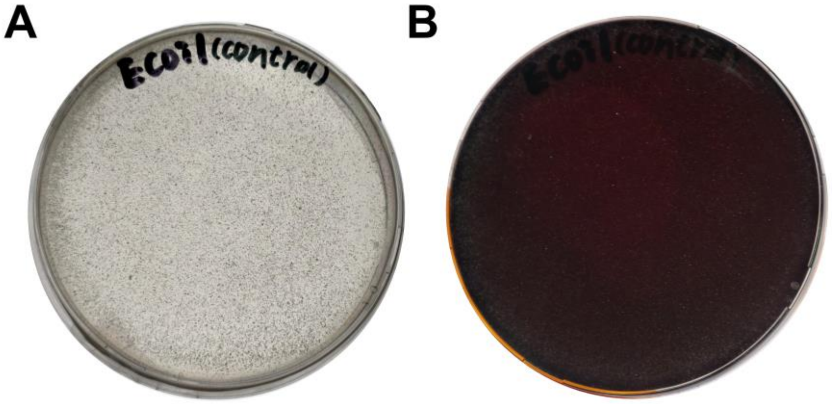

Add Figure S4

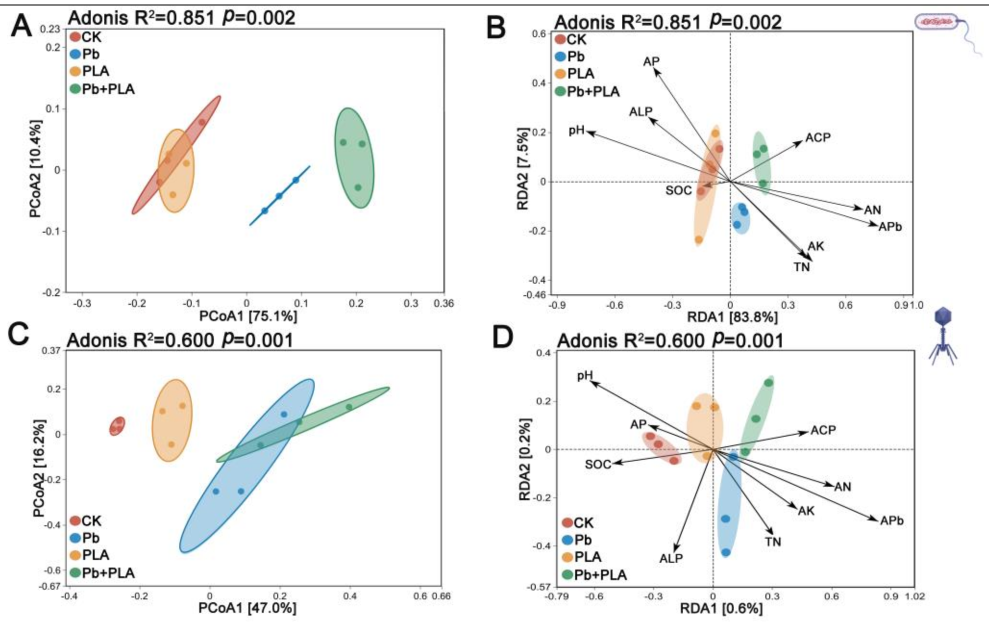

Revised_Figure S2, Changes The axis values at the endpoints have been displayed uniformly across all panels, and the font size has been increased. Additionally, the layout of the legends has been reorganized to ensure a clean presentation.

Reviewer #1 (Remarks to the Author):

The present study is well-written and eloquently describe an underexplored subject in terrestrial microplastic and heavy metal pollution. The impact of viruses on the metabolization and adaption to emerging pollutants builds a novel perspective and add substantial value to the existing knowledge. The addition of experimental data on biodegradation is a great indication for the quality of the work and I personally appreciate these data in times were sequencing data are very dominant in the plastisphere research, even though the applied method has certain limitations (s. below).
Despite my recommendation for publishing this great work I would ask the authors to generally give more explanations on the extent and impact of the two applied pollutants Pb and PLA and their projected impact in the future and why a co-contamination is likely in which types of environments?
(**Response**: Thank you for this important suggestion. In revised Introduction part, we try to give more explanations of the pollutants Pb and PLA **(Line 90-92, Line 128-134)**
At certain paragraphs especially in the discussion the authors should add more cross references for their own data.
My personal expertise does not qualify me to comment too much on certain statistical analyses and the elaborated description of viral sequence evaluation.
In the following there are certain comments and suggestions that I kindly ask to work through before publication.

l. 16 Could use „ authors“ as there are two persons mentioned.
**Response:** Thank you for let us notice that, and it has been corrected **(Line 16)**
2.21,22 The statement appears to be very strong and I would disagree that Biodegradable microplastic and heavy metals are that often-co-occurring.
**Response:** Thanks for this important comment. We agree that the original wording overstated the frequency of co-occurrence between biodegradable microplastics and heavy metals. We have revised the corresponding sentence in the Abstract to clarify that such co-occurrence is context-dependent rather than ubiquitous. Specifically, we now emphasize that biodegradable microplastics and heavy metals may coexist in particular soil environments where plastic inputs overlap with metal contamination (e.g., agricultural systems, compost- or sludge-amended soils, and contaminated sites). **(Line 26-28:** Biodegradable microplastics and heavy metals can co-occur in soils due to overlapping anthropogenic inputs, including plastic mulching, compost or sludge amendment, and legacy metal contamination.)
3. 25 Would leave out “in a controlled trial”
**Response**: Thanks for the suggestion. “in a controlled trial” has been deleted in the original sentence **(Line 29).**
4. 42 Would suggest to add that PLA degrades extremely slow under environmental conditions and thus PLA microplastic pollution can be an emerging threat, even though it is biodegradable.
**Response:** We thank for this valuable suggestion. We agree that, despite being classified as biodegradable, PLA degrades extremely slowly under natural environmental conditions. We have

therefore revised the Introduction to explicitly acknowledge the slow degradation kinetics of PLA in soils and to emphasize that PLA microplastics may persist and represent an emerging environmental concern **(Line 90-92:** Despite being classified as biodegradable, PLA degrades extremely slowly under natural environmental conditions, and thus PLA-MPs pollution can be an emerging threat.)
5. 45 Could you elaborate more how this "passive sampling" influence heavy metal mobility.
**Response**: We thank the reviewer for this insightful comment. In the revised manuscript, we have clarified the mechanism underlying the "passive sampling" effect of microplastics. Specifically, we now explain that microplastics possess negatively charged surfaces and diverse functional groups, which promote the adsorption of heavy metal ions onto MP surfaces. Through this adsorption, microplastics can act as vectors that retain and redistribute heavy metals within soil systems, thereby influencing their mobility. This clarification has been added to the Introduction, and we also add the reference for explaining this **(Line 92-94:** After entering soil, MPs can act as passive samplers and vectors for heavy metals, as their negatively charged surfaces and diverse functional groups promote the adsorption of metal ions onto MP surface)
6. 79 I would rephrase the last hypothesis as it is counter-intuitive to label PLA degradation as disruption and would focus more on how the interplay of both contaminants acts in terrestrial ecosytems.
**Response:** We thank the reviewer for this insightful comment. We agree that the original hypothesis could be misleading by implying that PLA degradation itself acts as a disruptive or mitigating process. In the revised introduction part, we have rephrased the final hypothesis to avoid this interpretation and instead emphasize the interactive effects between Pb contamination and PLA microplastics on soil microbial–viral communities. Specifically, we now focus on how the co-occurrence of these contaminants reshapes soil microbiome and functional potentials, including viral AMGs related to nutrient cycling and PLA-associated metabolic processes. (**Line 161-165:** We hypothesized that Pb contamination would negatively impact soil microbiota diversity and function, affect virus-host associations, while the co-presence of PLA-MPs would adjust these Pb-driven effects, particularly by reshaping microbial-viral interactions and enriching viral AMGs associated with nutrient cycling and PLA-associated processes)
7. Remark to the introduction: I am missing information about what streams of contamination do you have in mind. Describe in which amounts PLA and Pb are entering soil systems to underline the importance of both pollutants. If possible, it would be helpful to give estimates of the quantity of pollutants that is expected to enter the environment. Either on a global scale or in the distinct region you have set the study.

**Response:** Thank you for this helpful comment, and agree the information about the contamination is very important for the research background. In the revised Introduction, we have expanded the environmental background to clarify both the major contamination streams and representative concentration levels of Pb and microplastics in agricultural soils. Specifically, we now cite national-scale assessments showing that Pb pollution is widespread in Chinese agricultural soils over the past three decades and more severe contamination in southern regions. In parallel, we have added large-scale estimates indicating that microplastic contamination in croplands has increased markedly over recent decades, with average concentrations rising from ~100 items kg^{-1} soil in 1980 to over 2,000 items kg^{-1} soil by 2018. We also note that polymer-specific data for biodegradable microplastics such as PLA in soils are currently very limited. This is mainly because most large-scale surveys of soil microplastics do not differentiate polymer types, and because the agricultural use of biodegradable plastic films is a relatively recent practice, resulting in a lack of long-term field measurements. Nevertheless, biodegradable plastic films, including PLA-based materials, are being actively promoted and increasingly adopted in Chinese agriculture. We have therefore emphasized in the revised Introduction that PLA inputs to agricultural soils are likely to increase in the near future, underscoring the importance of investigating the ecological impacts of PLA microplastics before their widespread accumulation occurs. **(Line 128-134)**
8. 86, 87 please make sure if kg soil for fresh/ wet soil or dry soil
**Response:** Thanks for this important clarification. In the revised manuscript (2.1 Soil Collection and Pot Experiment Design), we have specified that all Pb and PLA microplastic application rates are expressed on a dry soil weight basis. **(Line 170)**
**9.** 89 Is the agricultural soil in active agriculture or just arable land? As you describe the elimination of litter and stones it sounds like inactive agricultural soil.
**Response:** Thank you for pointing that. In the revised Supplementary Materials, we have specified that the soil was collected from an actively cultivated agricultural field. We also clarified that the removal of visible plant residues, leaf litter, and larger stones prior to sampling was a standard pre-treatment step to ensure soil homogeneity and to avoid interference from undecomposed organic debris, rather than an indication of inactive or abandoned land (**Supplementary Methods, Line 28-31**).
10. 125 Would appreciate to name the author of the method
**Response**: We thank you for this suggestion. In the revised manuscript *(2.4 DNA Extraction and Sequencing of Viral Fraction)*, we have specified the original reference for the ultracentrifugation-based virus collection method by citing the author **(Line 228:** Viruses were collected using the ultracentrifugation method as previously described by Szpara et al. [32];).
11. 139 de novo in italics
**Response**: Thank you for pointing this detail. "de novo" has been corrected in italics **(Supplementary Methods, Line 166)**.

12. 154-159 Could you elaborate more on how you combined the two software results and if the “High-confidence sequences” were from Virsorter or from CheckV.
**Response:** We thank you for this important clarification. In the revised manuscript *(2.5 Identification and Classification Annotation of Viral Sequences),* we have explicitly described how the outputs of CheckV and VirSorter2 were integrated. Specifically, high-confidence viral sequences were primarily defined based on CheckV quality assessment (high- and medium-quality contigs). VirSorter2 was then used as a complementary tool to recover additional putative viral contigs that were not retained by CheckV but exhibited strong viral signatures. By combining CheckV-based quality classification with VirSorter2 predictions, we aimed to improve the sensitivity of viral detection while maintaining stringent confidence criteria. And above clarification has been added to the Methods section **(Supplementary Methods, Line 174).**
13. 172 Would appreciate if you add a reference here.
**Response:** Thank you for this suggestion. We have added the reference for the RPKM normalization framework. Specifically, viral contig abundances were calculated based on read coverage normalized by contig length and total mapped reads, following the RPKM concept originally defined by Mortazavi et al. (2008) **(Line 324)**
14. 196 Please elaborate more on how you combined the different tools together for annotation
**Response:** Thank you for this helpful suggestion. In the revised Materials and Methods, we have clarified the annotation workflow by explicitly describing the sequential use and integration of VirSorter2, DRAM-v, and multiple functional databases. Specifically, viral genes were first predicted and classified using VirSorter2, followed by confidence scoring and preliminary functional categorization using DRAM-v. Functional annotations were then refined using an integrated multi-database approach (Pfam, UniProt, MEROPS, CAZy, and VOGDB), and annotations from different databases were integrated to assign functional categories.
15. Chapter 2.9 I would expect to have read the CarE identification in the results part and not in the M&M part. Would suggest to rephrase and focus less on this specific gene.
**Response:** We thank you for this insightful suggestion. We truly agree that the identification and selection of the carboxylesterase candidate represent a result rather than a methodological description. In the revised manuscript, we have restructured this section by moving the description of CarE identification and the rationale for selecting the representative gene to the Results section. The Materials and Methods section now focuses exclusively on the general screening strategy and experimental validation framework, without emphasizing the specific gene.
16. 229 I like the concept, but would suggest to make an additional chapter with the experimental validation of CarE as it would help the readers to follow.
**Response:** Thank you so much for this constructive suggestion. Following this advice, we have restructured the Materials and Methods section by separating the screening of putative PLA-degrading enzymes and their experimental validation into two independent subsections (**Sections**

2.9 and 2.10). The screening subsection now focuses on the bioinformatic identification of candidate enzymes, while the experimental validation subsection exclusively describes the heterologous expression and enzymatic assays. We hope this revision allows readers to more clearly follow the work here.

17.For some companies you give the city name for others you don´t. Please harmonize

Response: Thank you for point that. We already harmonized this in the revised version.

18. 235 Please give more information about the origin of PLA as well as particle size etc. and what exactly do you mean by emulsified PLA (Supplement)

Response: Thank you for this important comment. We have now clarified the origin, particle size of PLA microplastics in the Materials and Methods and Supplementary Information. Specifically, emulsified PLA was prepared following published procedures (Teeraphatpornchai et al., 2003) by dissolving PLA in dichloromethane, emulsifying the polymer by sonication in distilled water, and subsequently removing dichloromethane by incubation at 80°C. The resulting aqueous PLA emulsion was then used to prepare PLA-containing medium (final PLA concentration 0.1%, w/v). No surfactants or additional chemical emulsifiers were used. In Teeraphatpornchai's studies, emulsification of PLA has been employed as a methodological step to improve dispersion and increase the effective surface area of this hydrophobic polymer in aqueous culture systems, thereby enabling reproducible evaluation of microbial degradation rather than inducing chemical breakdown of the polymer itself. And about above explanation, we already added in the Supplement material **(Supplementary Methods, Line 54:** The PLA microplastics used in this study had particle sizes of 13–15 μm and a reported polymer purity of 99%, and were obtained from Dongguan Huachuang Plastic Chemical Co., Ltd. (Dongguan, Guangdong, China). **Supplementary Methods, Line 219-226:** The emulsification of PLA was performed to improve the dispersion and homogeneity of this hydrophobic polymer in aqueous culture media, thereby increasing its effective surface area and ensuring reproducible microbial access to the substrate, as described in previous studies**).**

Teeraphatpornchai T, Nakajima-Kambe T, Shigeno-Akutsu Y. 2003. Isolation and characterization of a bacterium that degrades various polyester-based biodegradable plastics. Biotechnology Letters, 25: 23-8. https://doi.org/10.1023/A:1021713711160.

19. 236 I like the staining idea! Is there a reference for it or have you come up with the idea by yourself? Could you briefly describe what exactly it is staining? Like PLA Monomers or something else?

Response: Thank you so much for like our idea. The iodine–potassium iodide (I_2–KI) staining method was not developed by ourselves but adapted from previously published studies that used I_2–KI staining to visualize polymer degradation halos on solid media (e.g., PE and PVA degradation assays). In these studies, iodine preferentially stains intact or residual polymeric substrates dispersed in the agar matrix, producing a dark background, while regions where polymers have been degraded remain unstained and appear as clear zones. In our study, this

approach was applied to emulsified PLA-containing agar. It should be clarified that I_2–KI does not stain PLA monomers or degradation products; instead, it stains residual polymeric PLA particles. Therefore, the appearance of a transparent halo reflects local depletion of polymeric PLA caused by enzymatic hydrolysis rather than direct staining of PLA monomers. We have added appropriate references accordingly. **(Line 497)**

20.247 Where parametric assumptions met for all displayed data or was it necessary to do other tests for certain data?

Response: Thank you for this important question. Prior to statistical analyses, all datasets were individually tested for normality (Shapiro–Wilk test) and homogeneity of variance (Levene's test). For datasets that met parametric assumptions, two-way ANOVA followed by Tukey's multiple range test was applied. We have clarified this procedure in the revised Methods section **(Line 504:** Before analysis, all datasets were tested for normality using the Shapiro–Wilk test and for homogeneity of variance using Levene's test.)

21. 259 Consider to make a separate chapter for RDA plot instead of having such an elaborated statistical analysis chapter

Response: Thanks for this helpful suggestion. In the revised manuscript, we have streamlined the Statistical Analyses section and separated the description of RDA into a distinct paragraph placed at the end of the section. This revision clearly distinguishes RDA from general statistical tests and community comparison analyses, while maintaining a concise structure without introducing additional subsections **(Line 591-595)**.

22. 269 I personally prefer to have one or two descriptive sentences before starting with the results part right away. Also, I would suggest highlighting that BOTH treatments with Pb have effects to make it more easy to grasp your information.

Response: We thank you for this helpful suggestion. In the revised manuscript, we have added introductory descriptive sentences at the beginning of **Section 3.1** to summarize the main focus and overall trends of the results. We also explicitly highlight that both Pb and Pb+PLA treatments (Pb-containing treatments) significantly affected soil properties **(Line 599).**

23. 277 Would suggest to reorder the results and focus for instance first only on macroscopic plant growth than contents and lastly the activities. Further please give a brief explanation what do you want to examine with MDA and POD activity or what you can retrieve from these data.

Response: Thank you for the suggestion. The Results section has been reorganized accordingly. We now first describe macroscopic buckwheat growth traits, followed by changes in plant Pb content and stress-related indicators, and finally physiological enzyme activities **(Line 639-643)**. And we add a brief explanation about the MDA and POD activity in Method section (***2.2 Determining Soil Physicochemical and Buckwheat Properties*** **Line 193-196)**

24. 288,289 What exactly are you describing with the sign "~"

Your bacterial nomenclature is outdated as for instance Proteobacteria are named Pseudomonadota now. Please adjust all names in the text and in the figures accordingly.

https://www.microbiologyresearch.org/content/journal/ijsem/10.1099/ijsem.0.005056
https://www.nature.com/articles/s41522-024-00494-9
check the work from Oren et al.

Response: Thank you so much for pointing this out. The symbol "~" was intended to indicate the range of relative abundances observed across samples. In the revised manuscript, we have replaced this notation with a clearer description using standard format (e.g., "ranging from X% to Y%") to avoid ambiguity **(Line 650-652).**

And we so thank you for highlighting that our bacterial nomenclature is outdated. We have carefully reviewed and updated all bacterial phylum names throughout the manuscript, including the main text, figures, and supplementary materials. Phylum nomenclature has now been standardized using the validly published ICNP names with the suffix "-ota", following the recommendations of Oren (2024) and the LPSN/NCBI taxonomy. A clarifying statement has been added to the Methods section to explicitly indicate the nomenclature framework adopted in this study **(Line 223:** Phylum names were reported using ICNP-valid nomenclature (suffix "-ota") following Oren (2024), LPSN, and NCBI taxonomy standards **Error! Reference source not found.).**

25. 307 I am not a virologist, just double check if there have been changes in the nomenclature for viruses in recent years

Response: Thank you for the notice. We carefully re-checked all viral taxonomic names against the latest ICTV updates, including the ratified taxonomy changes summarized by Simmonds et al. (2025). At the genus level, all reported taxa remain valid under the current ICTV framework. At the family level, we note that traditional families such as Podoviridae, Myoviridae, and Siphoviridae are no longer formally recognized due to the extensive reorganization of tailed bacteriophages within the class Caudoviricetes. However, these terms are still widely used in present metaviromic studies and are the default output of commonly applied annotation tools (e.g., VirSorter2, VOGDB). We therefore retained these names for consistency and comparability, and clarified this point in the Methods section **(Line 318-321).**

26. 311 there is a space missing between Fig. and 1D please recheck these minor typos in the entire manuscript.

Response: Thank you for point that. It has been corrected in revised version.

27. 316 I suggest to change the wording and do not use correlate as this is not well fitting in this context.

Response: Thank you for this suggestion. We have revised the wording to avoid the "correlate" and now describe the results as significant associations identified by envfit. (**Line 775:** Fitting all soil physicochemical variables using envfit revealed that pH and APb, AK, TN, and AN content were significantly associated with virome ordination)

28. 326 Could you explain why exactly you chose the phylum-family link pattern and not phylum order etc.?

Response: We thanks for your insightful question. Host assignments at finer taxonomic levels

(e.g., genus or species) are often uncertain due to limited reference genomes and the indirect nature of prediction methods, whereas higher-level groupings (e.g., order or class) may obscure ecologically meaningful differences. Using bacterial phyla and viral families therefore provides a stable and interpretable framework that has been widely adopted in previous virome studies (https://doi.org/10.1038/nature19094,https://doi.org/10.1038/s41564-018-0190-y, https://doi.org/10.1038/s41396-022-01188-w).
29. 330,331 I have never done these links by myself but I wonder if there is kind of a variation between your replicates as you do not give any value variation for different treatments. Would suggest to give a brief elaboration of what you want to show with these data.
**Response:** Thank you for this important question. The virus–host links reported in Table S9 were derived from pooled host prediction results across biological replicates within each treatment. Therefore, these values could be a little different, they represent cumulative frequencies of distinct phylum–family virus–host associations rather than replicate-level quantitative measurements, and measures of variation (e.g., mean±SD) are not applicable. Differences among replicates are implicitly reflected in whether specific links are consistently detected across samples. Accordingly, count-based non-parametric statistical tests (Scheirer–Ray–Hare and Fisher's exact tests) were applied to evaluate treatment effects. This clarification has been added to the revised Method section **(Line 510-514).**
30. 337 Not familiar with these types of analysis, but why using first a Scheirer-Ray-Hare test and here a Fisher test?
**Response:** Thank you for point this. These two tests were applied to address different analytical objectives. The Scheirer–Ray–Hare test, a non-parametric two-factor extension of the Kruskal–Wallis test, was used to evaluate whether Pb and PLA treatments had significant effects on the overall composition of virus–host links, while accounting for interaction effects. In contrast, Fisher's exact tests were subsequently applied to individual phylum–family virus–host links to examine whether specific associations differed significantly between treatments. And we use this two-step approach to distinguish between global shifts in virus–host association structure and potential changes in individual link types. About the above explanation, we already added them in revised Methods section (**(Line 510-514).**
31. 338 Why is this finding notably? Please elaborate why you highlighted these. Are you referring with Chordata to the macroscopic animal group because I have not found a bacterium phylum named Chordata? Please clarify
**Response:** Thank you so much for point this important comment. Here, Chordata refers to host phylum annotations derived from computational host prediction tools rather than implying viral infection of macroscopic animals. We agree that highlighting these rare associations as "notable" was misleading. Host assignment in this study was based on computational inference using CHERRY and PHP. As previously reported in viromics studies, computational host prediction remains challenging because most environmental viral sequences lack direct host information,

and reliance on sequence-based inference can lead to uncertain or spurious host assignments (https://doi.org/10.1016/j.coviro.2021.05.003). This likely explains the occasional assignment of viral contigs to non-bacterial phyla. To ensure a conservative interpretation aligned with the focus of this study, downstream analyses and discussion were restricted to virus–bacterial host associations. Host predictions involving non-bacterial phyla (e.g., Chordata) were therefore excluded from interpretation. And we clarified it in the revised Methods section (***Section 2.7, Host Predictions…Line 389***). Importantly, this revision does not affect any statistical analyses or conclusions, as these rare associations were not included in the Scheirer–Ray–Hare or Fisher's exact tests.
32. 341 Why should a virus interact with a different host when the treatment changes?
**Response:** Thank you for this insightful comment. We did not intend to imply that viruses actively switch hosts in response to Pb or PLA treatments. Rather, our results indicate that the dominant virus families and host phyla were consistently detected across all treatments, suggesting that virus–host associations were largely structured by shared community members and remained stable under contamination. Short-term environmental perturbations are unlikely to alter viral host range, which is typically constrained by long-term co-evolutionary relationships. We have revised the Discussion (***4.3 PLA Mitigate Pb-Induced Disruptions in Rhizosphere Viral Communities and Host Interactions***) to clarify this.
33. 346 if you highlight level2 category please make sure to explain what is the difference to other levels and why you use level2
**Response:** Thank you for this comment. Bacterial and viral functional genes were summarized at KEGG level 2 to enable comparison of specific biogeochemical processes (e.g., carbon, nitrogen, and phosphorus metabolism), which provides an appropriate balance between functional resolution and ecological interpretability. In contrast, given the relatively limited number and uneven functional distribution of virus-encoded auxiliary metabolic genes (AMGs), their functional annotations were summarized at KEGG level 1 to capture broad functional categories while avoiding excessive fragmentation and instability at lower pathway levels. We have clarified the rationale for using different KEGG functional levels in the revised Methods section **(*2.8 Bacterial and Viral Gene Annotation Line 462-465*).**
34. 347 Is the list done only with Bacmet or also manually curated or could you name a reference for this? I just wonder as I am not aware how the function "Drug resistance: Antineoplastic" and others are related to Pb tolerance.
**Response**: Thank you for raising this important point. We apologize for the lack of clarity in the original wording. The functional categories shown in Table S10 originate from different annotation frameworks. KEGG level 2 categories (including "Drug resistance: antineoplastic" and other pathways) reflect broad functional classifications assigned by the KEGG database and do not imply a direct association with Pb tolerance. These categories represent general cellular response and stress-related pathways commonly annotated in environmental metagenomes. In

contrast, Pb resistance genes were independently identified using the BacMet database, Pb resistance was therefore analyzed and interpreted separately from KEGG-based functional categories. We have revised the corresponding text in the manuscript. Regarding the reference literature, Zheng et al. (https://doi.org/10.1038/s41396-022-01188-w) employed a functional annotation strategy for bacterial and viral communities that shares some similarities with our analysis.
35. 350 I am concerned about the label "relative abundance" as in Table S10 a treatment would have more than 100% relative abundance. Please clarify what the values in Table S10 are meaning and what they mean for your results. It is up to you, but I would suggest to highlight some Pb/ PLA related damage potentials to your description. For instance, "Replication and repair" is dramatically increased in Pb treatments as well as Aging. I think you mention them later in the discussion, too.
**Response:** Thank you for this helpful comment. We would like to clarify that relative abundances are shown in Fig. 3A, where KEGG functional gene profiles are visualized as relative abundances to illustrate compositional shifts across treatments. Table S10 does not present relative abundances. Instead, it reports F statistics derived from two-way ANOVA, which quantify the strength of PLA, Pb, and interaction effects on different functional gene categories. We added the note in Table S10.<br>Following the reviewer's suggestion, we now also emphasize biologically meaningful Pb- related potentials. In particular, functional categories related to replication and repair showed a strong Pb-associated effects, which is added in the revised Result section (***3.5 PLA and Pb Drive Divergent Functional Responses of Rhizosphere Bacterial and Viral Communities Line 891***).
36. 363 I think you could mention that overall, the effects of the treatments on viral functions appear to be much lower compared to the changes induced in bacterial function, right?
**Response:** Thank you, and it is a very good suggestion here. The functional responses of viral communities were weaker than those observed for bacterial communities. And we have revised the Results section to explicitly highlight this pattern **(Line 900:** Overall, the effects of the treatments on viral functions appeared smaller than those induced in bacterial communities.).
37.382 Any hints of enhanced quantities in comparison to non-PLA treatments?
**Response:** Thank you for this insightful question. In this study, the genes were identified based on contig-level functional annotation rather than quantitative abundance metrics. While we did not quantify CarE genes using read-based abundance measures, we observed an enrichment in their occurrence under PLA-containing treatments. Specifically, five out of seven putative viral CarE genes were detected exclusively in PLA and Pb+PLA treatments, whereas only two were found in non-PLA treatments (CK and Pb) (Table S12).
38.389 If talking about multi-database name them or at least add references.
**Response:** Thank you for the suggestion. The section was modified now **(Line 978)**.
39. 386 Maybe you could rename the long gene names for simplification and define them in the

supplement whenever useful.
**Response:** Thank you for this helpful suggestion. In the revised manuscript, we simplified the long gene identifier P9222_28545 by introducing the abbreviated name P92 for readability. The abbreviated name is used consistently throughout the main text **(Line 980).**
40. 397 Is it really the CarE enzyme activity. As I understood it, your enzyme assay is quite unspecific, thus I would argue to rephrase it.
**Response:** Thank you so much for pointing this. Although the activity was measured using a commercial carboxylesterase assay kit, we agree that the assay reflects general esterase activity rather than CarE-specific catalysis. We therefore revised the text to avoid over-specification, while retaining "carboxylesterase" as an annotation-based identity supported by conserved motifs and database classification. While the assay here is general, the significant increase in esterase activity in the *E.coli*+P strain (compared to the control) directly correlates with the clear degradation zones observed in the PLA-plate assay (Fig. 5C). This combined evidence suggests that the P9222_28545 gene encodes an enzyme with esterase function capable of degrading PLA **(Line 987-992).**
41. 414 S. above please explain more what POD activity indicates
**Response:** Thank you for this suggestion. And we sorry for missing this part in result section, and we already added the POD activity in revised version **(Line 643).** And we have expanded the explanation of POD activity in the Discussion **(Line 1053-1055).**
42. 417 I think it is even more fascinating that, the Pb content in PLA+Pb in soil is around 4,5% higher than in the Pb only incubation, though in the plant the Pb content is 9% higher in PLA+Pb than in the Pb only incubation. This further underline your hypothesis, that PLA MP enhances Pb mitigation. Overall, you are missing some data references in this part of the discussion, please add cross references to your data
**Response:** We thank for this insightful comment. Following this suggestion, we have revised the Discussion to explicitly cross-reference our quantitative results. Specifically, we now highlight that soil Pb concentrations in the PLA+Pb treatment were only slightly higher than in the Pb-only treatment (Table S2), whereas Pb accumulation in buckwheat tissues increased to a greater extent under combined PLA-MPs and Pb exposure (Table S4) **(Line 1052).**
43. 452 Is there anything known about virus scavenging on MP surfaces and thus shadowing effects?
**Response:** Thank you for raising this interesting point. There were some researches about the MPs and virus interacting (https://www.frontiersin.org/journals/microbiology/articles/10.3389/fmicb.2024.1433724/full,https://www.nature.com/nature-index/article/10.1016/j.watres.2022.119115), but most of them focus on the aquatic environment, while direct evidence for virus scavenging on MPs surfaces in soil systems is currently lacking. Based on the present study, we therefore consider that MPs may act as potential carriers of viral particles and could modulate viral responses to co-occurring

pollutants. We have revised the Discussion to explicitly acknowledge this possibility, while clearly stating that such mechanisms remain speculative and require targeted experimental validation. These clarifications have been incorporated into the revised manuscript. **(Line 1121-1123)**
44. 457 Maybe a bit off topic, but do you have any information about the vitality of the bacterial community when coping with such high Pb concentrations? Something like live-dead staining etc.? Might be useful to check for these and correlate with lytic and temperate viral lifestyles.
**Response:** Thank you for this thoughtful suggestion. We agree that direct measurements of bacterial vitality (e.g., live–dead staining) could provide valuable insights into host physiological status under high Pb stress and its potential links to viral lifestyle strategies. And in the present study, however, bacterial viability was not directly assessed, as our primary focus was on virome structure, viral lifestyle prediction, and functional potential inferred from metagenomic and viromic data. Notably, despite the relatively high Pb concentration, we did not observe a shift toward temperate viral lifestyles in the Pb or Pb+PLA treatments. This may indicate that the bacterial community retained sufficient metabolic activity to support lytic replication, or that Pb stress alone did not strongly favor lysogeny under the experimental conditions applied. Indirect support for this interpretation comes from the persistence of dominant bacterial taxa, and the absence of a collapse in overall bacterial diversity. We agree that integrating direct measurements of bacterial vitality and stress responses, and correlating them with viral lifestyle dynamics, would be an important avenue for future research.
45. 474 I disagree with your statement of PLA buffering Pb disruptive effects, as the total number of links was as low in PLA+Pb as in Pb only and just the distinct link number is changed. Further, I would ask to tone down the statement of “PLA contributing to ecosystem resilience” as this might lead to wrong conclusions when focusing on the entire ecosystem.
**Response:** We thank you for this important clarification. We agree that our original wording ay moverstated the extent to which PLA buffered Pb-induced effects on virus–host interactions. While PLA altered the number of distinct phylum–family link types, the total number of virus–host links remained similarly reduced under Pb and Pb+PLA treatments. We have therefore revised the text **(Line 1154).**
46.485 Please rephrase as hard to grasp what you want to say. And the Cadmium came a bit out of nowhere, please write the full name as this can easily be misunderstood as typo.
**Response:** Thank you for this comment and we agree that the original sentence was unclear. We have revised the text to explicitly spell out cadmium (Cd) and to clarify the comparison being made. The revised sentence now clearly distinguishes between the previous Cd+PLA study and our Pb+PLA results **(Line 1161-1163).**
47.491 add crossference to your data
**Response:** Thank you for pointing this. We already added crossference to corresponding data **(Line: 1166).**

48. 500 Would suggest to screen the literature if there are reports on N-cycling and Pb contamination.
**Response:** Thank you for the suggestion. We have revised the discussion section to incorporate relevant researches that Pb contamination can affect soil bacterial N-cycling genes **(Line 1190-1193).**
49. 508 Would rephrase “viral N metabolism” as this would indicate a more active role of viruses.
**Response:** We thank the reviewer for this helpful suggestion. We have revised the text to replace “viral N metabolism” with more precise phrasing that emphasizes viral AMGs associated with nitrogen-related metabolic pathways **(Line 1199).**
50. 528 I would tone down the paragraph as you do not have direct links to PLA degradation and you might overinterprete your esterase activity assay. I suggest to either convincingly argue that the assays are indeed indicating PLA degradation or be less enthusiastic about your conclusion. As the halos might be induced by defragmentation and not by mineralization. You have not provided information about actual monomers/ CO2 derived from PLA and not mentioned the content of small oligomeric PLA fractions or changed molecular masses. According to Sander et al. Biodegradation should be solely defined as the conversion of Bioplastic to CO2 or CH4.<br>Overall, I would like to have some hypothesize-like explanations why viruses should have an “interest” in enhancing PLA degradation in rhizosphere soil.
**Response:** Thank you for this constructive comment. We agree that our assays do not provide direct evidence for complete PLA biodegradation. We have therefore toned down the discussion to avoid overinterpretation. Accordingly, we revised our conclusions to emphasize that virus-encoded AMGs are unlikely to function as independent degraders of PLA. Instead, our data support the interpretation that these AMGs may indirectly support host metabolism under Pb and PLA stress by modulating carbon utilization potential. To address why viruses might have an “interest” in such processes, we now pointed that the addition of PLA may represent a selective pressure shaping virus-encoded AMGs. This interpretation is grounded in the enrichment of carbon utilization– and carboxylesterase-related AMGs in PLA-containing treatments (Fig. 3C; Table S12). Meanwhile, we also point that in future research, we want to elucidate the mechanistic pathways by which viruses regulate the biodegradation of microplastics within complex soil ecosystems ***(4.5 Virus-Encoded AMGs Reveal PLA Degradation Potential Under Pb and PLA Co-Contamination,*****Line 1222-1234).**
51. 591 rephrase as you have not done any toxicity assays in the study and the grammar is poor.
**Response:** Thank you for this important comment. We have revised this section to remove references to “hazardous substances”, and rephrased the text to focus on microbial functional responses and virus-mediated processes under PLA and Pb co-contamination **(Line 1300).**
52. Figure 1, I would ask to not make the 20 most abundant genera having a total relative abundance of 1, but to adjust them in a way that the reader can see what is their proportion on the total relative abundance. So either add “others” or adjust the y-axis for improved visibility.

The labeling is very small, please use larger font size.

Response: Thank you for this helpful suggestion. We have revised Figure 1 accordingly. Specifically, the stacked bar plots are no longer normalized to sum to 1 for the displayed taxa only. In addition, the font sizes of axis labels, legends, and annotations in Figure 1 have been increased to enhance readability. These changes are reflected in the revised Figure 1.

53. I am wondering why you have not indicated the phylum Deinococcus-Thermus in figure 2 and not having it in figure 1. Is there a missing "others" bar or something similar? Figure 2, Very small font size, please rearrange and increase visibility.

Response: Thank you for this observation. In Figure 1, we only displayed the top 10 most abundant phyla to maintain clarity, and Deinococcus-Thermus was not included as its abundance fell outside this range. Furthermore, we would like to clarify that our virus–host association analysis was conducted at the whole-community level, rather than being limited to the top bacterial phyla. As described in our Methods (**Lines 334**), we utilized CHERRY and PHP with stringent threshold filtering to ensure high-confidence host assignments. All viral contigs ≥ 1 kb with reliable bacterial host annotations were retained for downstream analysis. So, the reason Deinococcus-Thermus did not appear in Figure 1 was solely due to its relative abundance ranking outside the top 10, but it was not excluded from our computational host-prediction information.

Regarding Figure 2, we have rearranged the layout and increased the font size for all labels (including viral lifestyle, family, and host phylum) to ensure better visibility and professional presentation, as suggested.

54. Figure 3, Very small font size at some sections. Consider making two figures and increase font size substantially. In 3C Would suggest to order your pie plot from highest percentage to lowest, as this unordered visualization makes it counterintuitive to grasp the information. 3A Would suggest to put the Standardized gene abundance bar in the lower-right hand corner of the Heatmap. Further highlight the KEGG Level 1 categorization more (make the bars bigger for instance) 3C Include x-axis legend

Response: Thank you for these helpful suggestions. We have revised the figure accordingly to improve readability and data presentation. Specifically, we split the original Figure 3 into two separate figures to substantially increase font sizes and improve overall visual clarity. In panel 3A, the standardized gene abundance color bar has been repositioned to the lower-right corner of the heatmap, and the KEGG level 1 categorization has been more prominently highlighted by enlarging the corresponding bars. In panel 3C (now Figure 4), the pie chart has been reordered from the highest to the lowest percentage to facilitate intuitive interpretation, and an x-axis legend has been added to all bar plots.

55. Figure 4, Increase the font size and consider putting B and C below the plasmid map to increase visibility. Further, I kindly ask to add also a picture of a plate with a non-recombinant E. coli with and without staining to clarify the halo formation. With bigger size for the images would be appreciated.

Response: Thank you for this helpful suggestion. We have revised Figure 4 (now as Figure 5), and specifically, the overall font size has been increased, and panels B and C have been repositioned below the plasmid map. And following the recommendation, we have added representative images of plates inoculated with non-recombinant *E. coli* with and without I_2–KI staining to the Supplementary Information (Fig. S4, also can be seen below). The PLA-selective medium used in this assay contains emulsified PLA as the sole carbon source **(Supplementary method. <u>Line 213</u>)**. As shown in the newly added images, the non-recombinant *E. coli* did not exhibit visible growth or halo formation after incubation at 37 °C for 72 h, either before or after staining. This confirms that the host strain cannot utilize PLA under the applied conditions and that halo formation is not caused by background growth or staining artifacts. We have also increased the image size and adjusted the layout to improve clarity, as suggested.

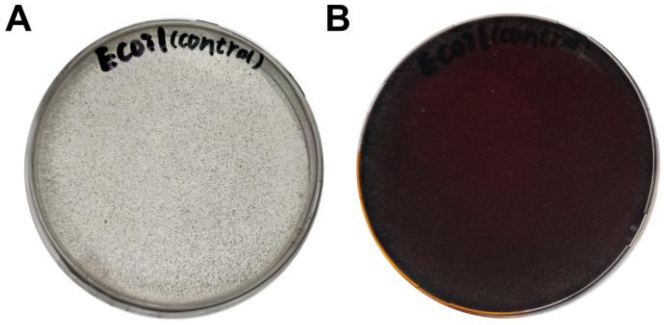

56. Supplement. Note: Is the Pb content of the tested soil in a normal range or rather high? If it has something like a Pb priming the results might be influenced.

Response: Thank you for raising this important point. According to the *Risk control standard for soil contamination of agricultural land* (China's soil environmental quality standard, GB 15618–2018) https://www.mee.gov.cn/ywgz/fgbz/bz/bzwb/trhj/201807/t20180703_446029.shtml, the risk screening value for total Pb in agricultural soils with pH 6.5–7.5 is 120 mg kg^{-1}. In our study, the background Pb concentration of the collected soil was 33.36 mg kg^{-1}, which is far below this threshold. Therefore, the background Pb levels of the soil sample are unlikely to have caused the Pb priming effects. And this information has been added in the **Supplementary Material, <u>Line 38-42</u>).**

57. Please clarify what exactly do you consider rhizosphere soil and how you obtained it. If available add a picture. Have there been separation methods to fractionate roots from soil or have you mixed root material together with surrounding soil etc.

Response: Thank you for pointing this. When harvest, buckwheat plants were carefully removed from the pots, and loosely adhering soil was gently shaken off. Soil particles tightly attached to the root surface were then carefully collected using sterile tweezers and defined as rhizosphere soil (see the picture below, please), and these samples were exclusively used for metagenomic and viromic sequencing. Other soil physicochemical properties were determined using bulk soil

collected from the root-surrounding zone. This bulk soil was not tightly attached to the roots but was distributed in close proximity to the root system. Above methods were following established protocols (Phillips and Fahey, 2006, https://doi.org/10.1890/0012-9658(2006)87[1302:TSAMAI]2.0.CO;2). We already supplied and clarified these information in the **Supplementary Material** <u>**Line 72-77.**</u>

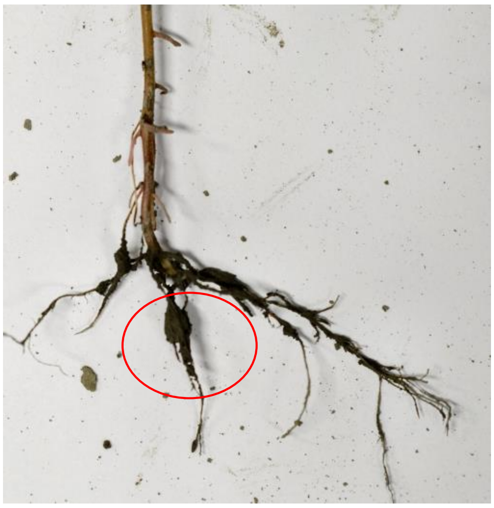

58. In the paragraph "determining soil properties" is a space missing before "[3]"

Response: Thank you for pointing that. We already checked and corrected it.

59: The plant lead content examination should be described more elaborately and also why an acid digestion is necessary and add a reference.

Response: Thanks for the suggestion. The method of plant lead content examination was supplied in **Supplementary Material** <u>**Line 87-91**</u>, and we also add the reference.

60. In general please be more elaborate on the whole degradation assay and what you can retrieve from the results and what limitations do you face

Response: Thank you for this valuable suggestion. We agree that a clearer description of the degradation assay is essential. In response, we have substantially expanded the methodological description of the PLA degradation assay and carboxylesterase activity measurement in the **Supplementary Methods (**<u>**Line 210-246**</u>**)**. We further clarified what can be inferred from these assays. The halo formation on PLA-containing plates and the increased esterase activity together provide functional evidence for enhanced ester bond–hydrolyzing capacity associated with the expressed gene, supporting its annotation as a putative carboxylesterase. However, we explicitly acknowledge the limitations of these approaches: the assays do not directly quantify PLA mineralization, nor do they measure PLA-derived monomers, oligomer size distributions, molecular weight changes, or CO_2/CH_4 production. Accordingly, the results are interpreted as indicating potential involvement in the initial hydrolytic or depolymerization steps of PLA, rather than complete biodegradation sensu stricto.

61. Figure S2, Sometimes the end values of the axis were mentioned like 0.72 and sometimes

not. Please harmonize. Further, the font sizes seem to be different. Please harmonize. Increase font size in general. Please double check if the axis in S2D are correctly labelled as I think 0.2% and 0.6% appear to be quite low and if this is correct please mention the low coverage of the analysis in the text.
**Response:** Thank you for these detailed suggestions. We have harmonized the axis ranges and tick labels across all panels in Figure S2. And we carefully checked the axis labels in Fig. S2D and confirm that the reported variance explained by the RDA axes (RDA1≈0.6%, RDA2≈0.2%) is correct. To address this point, we have now explicitly noted in the text **(Line 769)** that the low explanatory power reflects the high complexity of soil viromes and the limited ability of measured environmental variables to capture viral community variation.
62. Table S10 /S11, I personally do not like the table as mentioned before. It is not clear what the values are meaning and I would suggest to add CK values to simplify the understanding of the data.
**Response:** Thank you for this constructive comment. And we appreciate the suggestion to include CK values to facilitate data interpretation. However, Table S10 summarizes the results of a two-way ANOVA, in this framework, CK is inherently included as the reference level of the model rather than as an independent value. For our study, Table S10 was designed to provide the statistical support for the functional patterns shown in Fig.3. Specifically, Fig.3 visualizes the direction and magnitude of functional shifts across treatments, while Table S10 reports the corresponding two-way ANOVA results, identifying whether these shifts were driven by PLA, Pb, or their interaction. To improve clarity, we have expanded the table notes to explicitly link Table S10 with Fig. 3 and to clarify the meaning of the reported F statistics. For Table S11, we have added explanations to emphasize that the absence of significant differences in AMG counts indicates that treatment effects were mainly reflected in functional composition rather than absolute gene numbers.

Reviewer #2 (Remarks to the Author):

Attached accept with minor revisions

Reviewer #3 (Remarks to the Author):

1. Line 86: The authors should clarify the rationale for selecting the applied concentrations of PLA microplastics and Pb. In particular, a concentration of 2 g kg^{-1} appears relatively high. Please discuss whether these levels are environmentally relevant and to what extent the observed effects may reflect high-dose responses rather than conditions representative of real agricultural soils. The implications for field relevance should be addressed.
**Response:** Thank you for this important comment. The applied concentrations of PLA microplastics (2 g kg^{-1} soil) and Pb (2 g kg^{-1} soil) were intentionally selected to represent an elevated exposure scenario rather than average background levels in agricultural soils. The Pb concentration was chosen to induce clear microbial and viral responses while remaining below levels causing acute phytotoxicity. We acknowledge that these levels are higher than typical background concentrations; however, they were applied to enhance signal detection and to facilitate mechanistic exploration of microplastic–metal interactions, particularly virus-mediated functional responses, within a controlled pot experiment. Accordingly, we have clarified this in the revised Supplementary Methods, and we add correlated references to support the designed concentration (**Supplementary Methods, Line 47-52).**
2. Line 88: The rationale for selecting buckwheat as the model plant should be explained more clearly. Specifically, the authors should justify its relevance in terms of human diet, agricultural importance, and potential implications for public health or food security.
**Response:** Thank you for the suggestion. We agree that clarify the reason why choose buckwheat is important for the research, here, we add the explanation in introduction section **(Line 134-138).**
3. Line 92: The authors should briefly explain what each measured soil, plant, and biochemical indicator represents and its ecological or biological implications, rather than simply listing the parameters. This clarification should be applied consistently throughout the Methods section to improve methodological transparency.
**Response:** Thank you for this helpful suggestion. We have revised the Methods section to briefly explain the ecological or biological relevance of each measured soil, plant, and biochemical indicator, rather than only listing the parameters **(Line 177-197).**
4. Methods section (general): The Methods section is overly detailed. The authors are encouraged to retain only essential methodological information in the main text and move extensive procedural details to the Supplementary Materials, in line with the journal's formatting and readability expectations.
**Response:** Thank you for the suggestion. Following your advice, we have streamlined the

Methods section by retaining only the core experimental design and essential workflows in the main text. Primary Methodological Information: Descriptions of key steps, such as the overall bioinformatic pipeline, core assembly strategies, and major taxonomic annotation tools, remain in the main text. And the detailed information regarding DNA extraction, library preparation protocols, specific software parameters, and nomenclature standards (e.g., ICNP phylum names) has been moved to the Supplementary Methods.
5. Results section: P-values should be consistently formatted as lowercase italic p throughout the Results section.
**Response:** Thank you for your comment. We have standardized the formatting of *p*-values throughout the text and figures.
Results vs. Discussion:<br>6. The authors should more clearly separate the Results and Discussion sections. The Results should be limited to objective descriptions of the data without mechanistic interpretation, while the Discussion should avoid repeating experimental results. In the current version, there is substantial overlap between these sections, with repeated descriptions of results appearing in the Discussion.
**Response:** Thank you for this important suggestion. In the revised manuscript, the Results section now presents the data without bias or speculation. Conversely, the Discussion has been restructured to interpret these findings in the context of broader environmental impacts, avoiding the repetition of specific values or figures already descritied.
7. Depth of Discussion (e.g. Section 4.1): The discussion remains relatively superficial. For example, in Section 4.1, although comparisons with previous studies are provided, there is limited in-depth analysis of why the observed results occurred and what factors may explain similarities or discrepancies with earlier findings. A deeper mechanistic and contextual interpretation is needed.
**Response:** Thank you for the suggestion. In the revised discussion section, we try to point deeper mechanistic from the result. And we think that the revised version now offers a much clearer and more profound understanding of the ecological implications of Pb and PLA-MPs in terrestrial ecosystems.

Review Report:

Introduction: Well elaborated with few minor formatting mistakes

Few minor formatting mistakes; Page 6; line 180-187

Line-239-240

Line387-390

Methodology: formatting mistakes

Results section: line 479-483.

Line numbering is not clear and some results in section PLA and Pb Reshape Rhizosphere Viral Community are not readable.

Discussion: It will be better if add some more recent references about virus related biodegradation genes and enzyme work against pollutants.

Accept with minor revisions

Response: We sincerely thank you for the careful evaluation of our manuscript and for the positive assessment of its overall quality. We are pleased that the manuscript is considered suitable for publication with only minor revisions. Below, we address each comment in detail.

For formatting mistakes: The minor formatting issues have been carefully checked and corrected in the revised manuscript.

For Methodology: We have carefully reviewed the Methods section and corrected the reported formatting issues. In addition, we improved the clarity and consistency of formatting throughout this section to enhance methodological transparency.

Discussion: Following the reviewer's suggestion, we have strengthened the Discussion by adding several recent.

Overall, all formatting issues have been addressed, and the manuscript has been carefully revised to improve clarity, consistency, and completeness. We thank you again for the constructive comments, which have helped us to further improve the quality of the manuscript.

Review Report:

Introduction: Well elaborated with few minor formatting mistakes

Few minor formatting mistakes; Page 6; line 180-187

Line-239-240

Line387-390

Methodology: formatting mistakes

Results section: line 479-483.

Line numbering is not clear and some results in section PLA and Pb Reshape Rhizosphere Viral Community are not readable.

Discussion: It will be better if add some more recent references about virus related biodegradation genes and enzyme work against pollutants.

Accept with minor revisions
